# Understanding the photochemistry of a crystalline push–pull norbornadiene photoswitch

**DOI:** 10.1039/d5sc07670f

**Published:** 2026-02-10

**Authors:** Federico J. Hernández, Jordan M. Cox, Jingbai Li, Steven Lopez, Rachel Crespo-Otero

**Affiliations:** a Department of Chemistry, Queen Mary University of London Mile End Road London E1 4NS UK f.hernandez@qmul.ac.uk; b Department of Chemistry and Chemical Biology, Northeastern University Boston MA 02115 USA s.lopez@northeastern.edu; c Hoffmann Institute of Advanced Materials, Shenzhen Polytechnic University 7098 Liuxian Blvd Nanshan District Shenzhen 518055 People's Republic of China lijingbai@szpu.edu.cn; d Department of Chemistry, University College London London WC1 H0AJ UK r.crespo-otero@ucl.ac.uk

## Abstract

Molecular solar thermal (MOST) materials store and release solar energy through light-induced reversible reactions involving molecular photoswitches. Solid-state crystalline MOST materials can offer higher energy densities and easier device integration than their liquid counterparts. However, their photochemical mechanisms remain poorly understood. Norbornadiene (NBD), which undergoes a [2 + 2]-photocycloaddition to form its photoisomer quadricyclane (QC), has been proposed as a candidate for MOST applications. We used multiconfigurational quantum mechanical calculations and non-adiabatic molecular dynamics to investigate the mechanism of a push–pull NBD-derivative, 1,5,6-trimethyl-2,3-dicyanonorbornadiene (TMDCNBD). This study demonstrates a cutting-edge multiscale ONIOM(QM/QM′) nonadiabatic molecular dynamics framework in TMDCNBD crystals. The crystal packing of TMDCNBD preserves molecular flexibility, enabling ultrafast [2 + 2]-photocycloaddition *via* energetically accessible *S*_1_/*S*_0_ conical intersections, with negligible exciton transport. Simulations predict product quantum yields of 57% for TMDCNBD and 37% for its metastable quadricyclane (QC) form, TMDCQC, which stores 0.36 MJ kg^−1^. This work demonstrates push–pull norbornadiene photoswitches are promising crystalline MOST candidates and establishes a transferable computational protocol for modelling ultrafast photochemistry in the solid state.

## Abstract 2

This work demonstrates that push–pull norbornadiene photoswitches are promissing crystalline molecular solar thermal materials and establishes a transferable computational protocol for modelling ultrafast photochemistry in the solid state.

## Graphical abstract

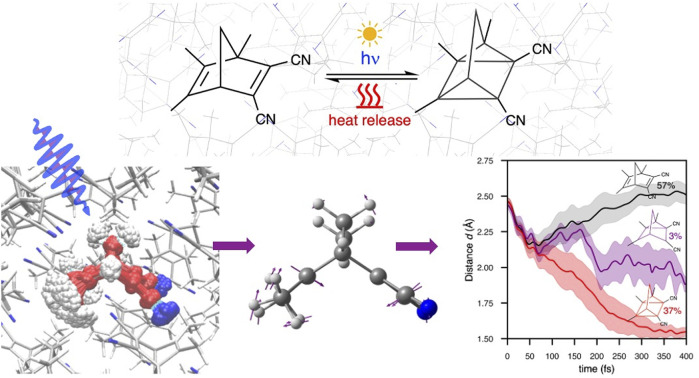

## 1. Introduction

Light-activated phenomena underpin applications in optoelectronic devices, sensors, and energy materials, among others. These processes occur in the condensed phase, where the environment plays an active role by either restricting the motion of excited molecules (the “cage effect”) or directly participating in the primary excited-state mechanisms.^1^ Molecular Solar Thermal (MOST) materials, also known as solar thermal fuels, represent an emerging technology that offers a promising and sustainable alternative for energy collection, storage, and on-demand release to the end user, all carried out by the same material.^1,2^ MOST materials rely on molecular photoswitches, *i.e.*, molecules that can reversibly switch between two or more chemical configurations upon exposure to light. Solid-state MOST materials can provide especially high energy densities and a high concentration of photoactive units per volume or mass. In this sense, different materials have been designed to undergo photo-induced phase changes, harnessing additional energy storage from the phase transition.^3–14^ Unlike their liquid counterparts, solid-state MOST materials are easier to integrate into devices. However, the development of solid-state MOST materials has lagged behind that of solution-based systems, despite several successful examples of crystalline MOST materials reported in the literature.^3–14^ One of the main factors hindering the rapid development of solid-state MOST materials is the added complexity of the solid environment, which challenges the rational design of efficient candidates. The nature, intensity and effect of intermolecular interactions, as well as effects such as accommodation of the photoactive unit within the environment, the crystal packing or electrostatics, on the photodynamics are still challenging to address and therefore remain majorly unexplored. A complete understanding of these phenomena at the atomic level is essential to optimise quantum efficiencies and support the design of new materials with tailored properties.

**Fig. 1:**
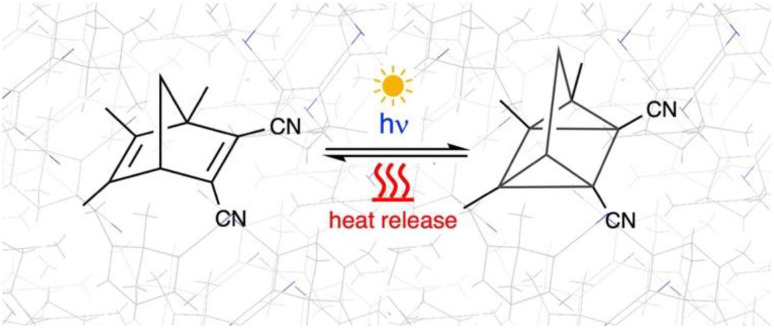
[2 + 2]-photocycloaddition of 1,5,6-trimethyl-1,3-dicyanonorbornadiene (TMDCNBD) schematised in the crystalline environment.

Norbornadiene (NBD)/Quadricyclane (QC) systems are among the most widely studied candidates for MOST applications, in which NBD undergoes a [2 + 2]-photocycloaddition to form the photoisomer QC (Fig. 1). The photodynamics of unsubstituted NBD/QC in the gas phase is ultrafast and has been well characterised both experimentally and computationally.^1,15–29^ However, unsubstituted norbornadiene is not a realistic model for practically relevant photoswitches due to its very low photoisomerisation quantum yield (QY ≈ 5%) and an absorption spectrum that is significantly blue-shifted relative to the solar spectrum reaching the Earth's surface.^1,15,16,30^ Derivatisation of NBD to improve its low isomerisation quantum yield and to red-shift its absorption spectrum has been a long-standing goal in the field.^31–41^ Nevertheless, there is very limited understanding of the factors that control the photodynamics of photoswitches, and even less is known about their behaviour in solid-state environments. Computational atomistic photochemistry offers a powerful strategy to deepen our understanding of these systems and to predict possible photoproducts. However, studying these processes computationally in the solid state remains extremely challenging due to the high computational cost of accurately modelling excited-state dynamics of chromophores in crystalline phases. The aim of this work is twofold: first, to investigate the photochemical reaction in a push–pull NBD derivative; and second, to implement readily computational strategies with focus on the investigation of photochemical reactions in crystalline organic materials.

Nonadiabatic dynamics, in particular trajectory-based methods such as surface hopping, can provide an insightful picture of light-activated processes for intra- and intermolecular processes.^42,43^ These methods have been successfully applied to understand excited state processes in organic systems from simple molecules to highly conjugated systems in complex environments.^44,45^ Describing photochemical and photophysical processes in the crystalline phase requires consideration of large number of molecules as well as the competition between radiative, nonradiative and reactive mechanisms. Additionally, determining whether these processes occur in a localised or periodic manner is important for identifying the most appropriate modelling strategies. The degree of localisation or delocalisation of excitations in crystalline systems is governed by the competition between exciton couplings and reorganisation energies.^42^ In many organic systems at room temperature, excitations tend to be localised on a few molecular units, with transport occurring *via* incoherent hopping. As a result, localised embedding methods such as quantum mechanics (QM)/molecular mechanics (MM) and QM/QM′ are often more suitable than periodic, delocalised approaches. This is particularly relevant for photochemical processes, where only a small number of molecules are typically excited by a laser pulse, and prolonged irradiation, often lasting hours, is required to achieve significant transformation between reactants and products.^46^

There is a myriad of interesting computational studies investigating photophysical processes such as charge and exciton transport and singlet fission in molecular crystals.^47–53^ Pragmatic computational approaches often implement reasonable approximations to reduce computational cost without compromising accuracy.^54^ Exciton models, where the excited states of molecular aggregates are constructed from the excited states of the individual molecular units are particularly common in this context.^47–52^ The classical path approximation and the neglect of back-reaction approximation (NBRA) have been employed to simulate charge transfer in large, condensed matter systems at the *ab initio* level.^55,56^ Several implementations of surface hopping under periodic boundary conditions have been developed, most of them based on NBRA.^57–60^ A recent approach combining excited-state gradients between Newton-X and CP2K software packages has also been published.^61^ Fully atomistic multiscale machine learning (ML/QM) methods, using neural networks trained on high-level quantum mechanical methods such as CASSCF and coupled to the semiclassical fewest-switches surface hopping (FSSH), have also been successfully applied to describe the various ultrafast singlet fission mechanisms occurring in the pentacene crystal,^62^ as well as to investigate aggregation-induced emission in hexaphenylsilole, tetraphenylsilole, and cyclooctatetrathiophene molecular crystals.^63^ An alternative approach based on the Fermi Golden Rule, has been used to investigate radiative and non-radiative rates in molecular crystals.^64,65^ These studies employed localised QM/MM methods to describe the excited states of the crystals, providing valuable insights into the photophysical processes of several organic materials displaying aggregation induced emission with applications in OLEDs and other related areas.

In contrast to the condensed phase photophysics, solid-state photochemical reactions are accompanied by substantial geometrical changes (*e.g.*, bond-breaking and -forming). Thus, the explicit modelling of excited-states nuclear positions is unavoidable. Initial works focused on the photoisomerization of azobenzene in solution using QM/MM and forcefields derived from nonadiabatic dynamics simulations.^66,67^ Some early attempts described photoisomerization reactions in a crystal of N salicylidene-2-chloroaniline and the proton transfer in crystals of 7-(2-pyridil)-indole by running a few semiclassical trajectories along with a mechanical embedding QM/QM′ (LR-TDDFT/DFT) scheme to compute the electronic energies.^68,69^ In this regard, semiempirical FOMO-CI combined with MM and semiclassical FSSH^70,71^ trajectories has been applied in the investigation of photoisomerization reactions in the solid state and in solution.^72–75^ For example, simulations of azobenzene-based self-assembled monolayers (SAMs) on Au(111) revealed that trans → cis isomerisation is suppressed in well-ordered structures due to steric hindrance, but can proceed efficiently in the presence of packing defects.^72^

Traditional methodologies based on NAMD in the gas phase can be exploited by approximating the environment using classical force fields (QM/MM) as available in well-known platforms such as Newton-X,^76^ SHARC^77^ among others. These programs have mostly been used to simulate photochemical reactions in solution. However, due to the specific characteristics of the crystalline phase, it is useful to develop tailored approaches for describing photochemical processes in molecular crystals. In this work, we leverage the fromage platform,^78–80^ which has been designed to provide tools for investigating excited-state processes in molecular crystals based on electrostatic embedding QM/QM′approaches, along with its interface with PyRAI2MD^62,81,82^ for nonadiabatic molecular dynamics (NAMD) simulations.

Herein, we use fully atomistic solid-state NAMD QM/QM′ multiscale calculations to study the [2 + 2]-photocycloaddition of 1,5,6-trimethyl-2,3-dicyanonorbornadiene (TMDCNBD, Fig. 1). We previously investigated the complete photodynamics mechanism that spanned the reaction coordinate from the photoexcited NBD in the gas phase to the formation of the corresponding photoproducts, predicting their respective QYs in very good agreement with the experimental results with complete active space self-consistent field (CASSCF) NAMD.^26^ We also pioneered a similar study for one of its derivatives, the 5,6-dimethyl-2,3-dicyanonorbornadiene (DMDCNBD) in the gas phase, to study the “push–pull” substituent effects (*i.e.* including electron donor and electron acceptor substituents within the same molecule) towards increased photoconversion quantum yields.^26^ The photoconversion is improved by essentially two major effects: the absorption spectrum is shifted to longer wavelengths, producing a better match with the solar spectrum reaching the Earth's surface^30^ and thus, increasing the probability of the photoswitch to absorb light; the other effect is the increase in the photoisomerisation QY towards the metastable isomer 5,6-dimethyl-2,3-dicyanoquadricyclane DMDCQC with respect to the value obtained for pristine NBD.^26^ Although the idea of the “push–pull” effect to improve the performance of NBD/QC molecular photoswitches has been under study for over a decade,^83–93^ there is still a limited understanding on the photodynamics of this type of MOST systems. Some previous experimental studies have incorporated modelling to assist in interpreting the photochemical behaviour of norbornadiene derivatives.^94,95^ However, these works rely on the analysis of the Franck–Condon region or are based on methods that are not suitable for accurately describing excited-state topologies and therefore do not provide a detailed mechanistic understanding of the underlying photodynamics. To the best of our knowledge, only one other theoretical study has investigated the photoisomerisation mechanism of two related push–pull NBD/QC photoswitches, and that work was carried out in the gas phase.^96^

In this work, we investigate the photodynamics of TMDCNBD in the crystalline phase by predicting and characterising its absorption spectrum, elucidating the pathways that lead to the formation of the principal photoproducts upon photon absorption, and quantifying their quantum yields through multiscale nonadiabatic molecular dynamics. We further examine how the crystalline environment shapes these photodynamic processes. Beyond the specific case of study, this work introduces a new strategy and openly accessible tools for probing ultrafast photochemistry and reaction mechanisms in molecular crystals. For the first time, we provide an atomistic, mechanistic understanding of the excited-state dynamics of TMDCNBD, accounting for both the multiconfigurational character of the involved excited states and the influence of the surrounding environment.

## 2. Results and discussion

### 2.1. Spectroscopic characterisation of TMDCNBD in the crystal

The equilibrium structure of TMDCNBD in the molecular crystal is very similar to the one in gas-phase. A RMSD = 0.12 Å is obtained when considering all the atoms and a RMSD = 0.08 Å when excluding hydrogens, suggesting that the crystalline packing does not significantly alter the ground-state geometry of TMDCNBD. This is rationalised based on the available space that each TMDCNBD molecule has to freely move within the crystal packing. The Voronoi volume and the van der Waals (vdW) volume for a TMDCNBD molecule in the crystalline structure give an estimation of the maximum available space in the crystal and the occupied space, respectively. We used the tools available in fromage^79^ to compute the ratio between the Voronoi and vdW volumes, namely, the volume index *V*_*i*_ (*V*_*i*_ = *V*_Voronoi_/*V*_vdW_), which gives an extent of the molecular flexibility. The *V*_*i*_ for TMDCNBD optimised in the crystal is *V*_*i*_ = 1.55, meaning that TMDCNBD has the 55% of its vdW volume to freely move in the crystal (See Fig. S1 in the ESI for a graphical representation of this).

**Fig. 2:**
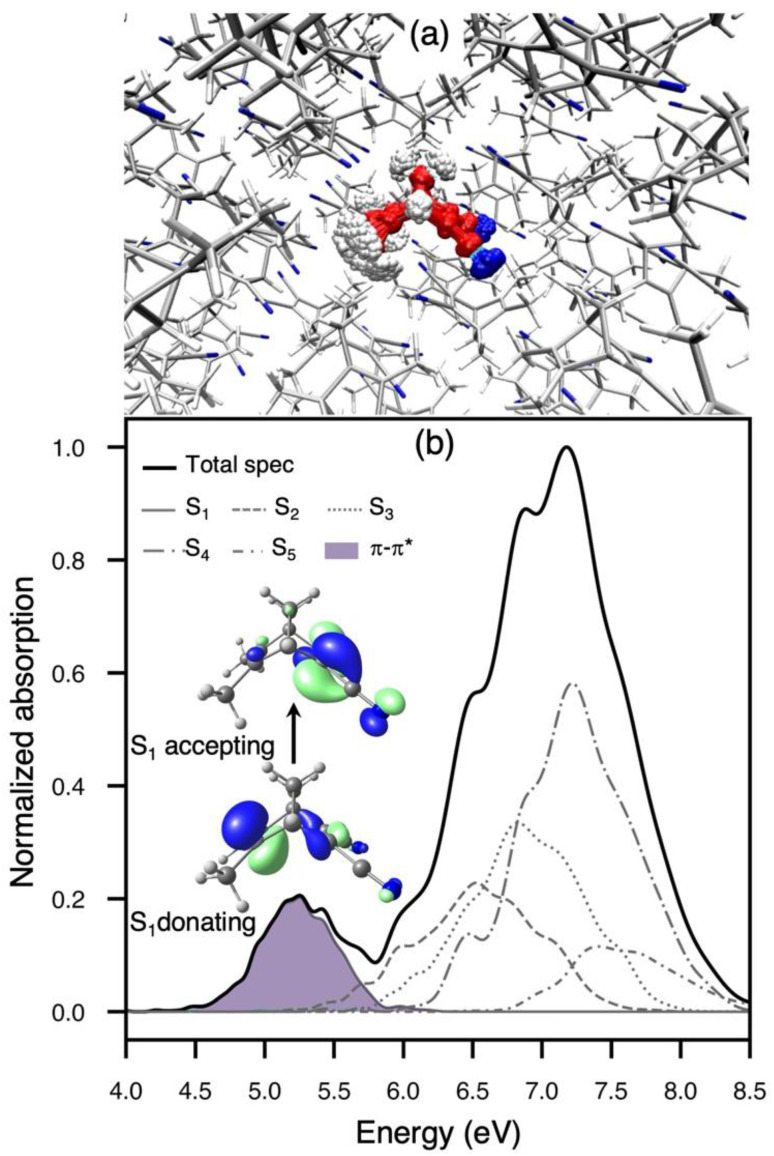
(a) Overlay of 200 Wigner-sampled initial conditions for the nuclear ensemble approach (b) Simulated absorption spectrum for the lowest two absorption bands of TMDCNBD in the molecular crystal computed with SA6-CASSCF[8,6]/ANO-S-VDZP and using 1000 Wigner-sampled configurations. The contributions of the lowest five adiabatic excited states to the total spectrum is shown. The purple shaded region in the lower-energy band represents the π–π* contribution to the first absorption band. *S*_0_ → *S*_1_ natural transition orbitals are also included.

To gain further insight into the spectroscopy of TMDCNBD in the crystal, we employed the nuclear ensemble approach (NEA)^97^ to calculate the absorption spectrum explicitly accounting for non-Condon effects, that are especially important for correctly describing the shape of the absorption bands and crucial to set the initial conditions for nonadiabatic dynamics following photoabsorption (Fig. 2).^54,97–99^ For this, we considered 1000 molecular configurations obtained from a harmonic Winger sampling as explained in Section 4. Considering the spectral region for the lowest energy bands, the absorption spectrum is characterised by two main absorption bands of markedly different intensities, and their peaks are predicted at 5.25 and 7.17 eV, respectively, at the SA6-CASSF[8,6]/ANO-S-VDP level of theory. Based on the results shown in Table S1 and on previous works,^26^ we expect the simulated absorption spectrum is overestimated by the SA(6)-CASSF[8,6] level of theory. However, in Section S2 of the SI, we show that the overall description of the band positions, band shapes, and absolute molar extinction coefficients is in very good agreement with experimental results measured in MeCN,^100,101^ as well as with the absorption bands calculated using NEA in combination with linear-response time-dependent DFT (LR-TDDFT) using the ωB97X-D^102^ functional and the aug-cc-pVDZ^103^ basis set together with implicit solvation for MeCN. This provides a quantitative comparison with experiment, as recently demonstrated for other complex photoswitches.^104^

The crystalline packing produces a significant red shift in the absorption transitions where the major shift is observed for the lower energy transition (Table S1 and Fig. S2 from the SI). A red shift of 0.4 eV is obtained when comparing the low-energy absorption band calculated in the crystal with respect to gas phase at the LR-TDDFT/ωB97X-D/aug-cc-pvDZ level of theory (Fig. S2). A similar shift of 0.4 eV and 0.34 eV is also obtained for *S*_1_ excited state when calculated with both SA6-CASSCF[8,6] and XMS(6)-CASPT2[8,6], respectively.

The lower-energy absorption band is well separated from the other one, and it is dominated by the *S*_1_ state with only contribution from a ππ* character with partial charge transfer (see the natural transition orbitals in Fig. 2b and results in table S1 and Fig. S3–S5 in the SI). A population analysis performed at the SA6-CASSCF[8,6]/ANO-S-VDZP level of theory shows that the partial charge transfer (CT) corresponds to 0.59e from the methyl to the cyano groups. A population analysis calculated at XMS(6)-CASPT2/SA6-CASSCF[8,6]/ANO-S-VDZP shows a CT = 0.65e and a natural populations analysis calculated with LR-TDDFT/ωB97X-D/aug-cc-pVDZ gives a CT = 0.55e supporting the assignment of the first absorption band. The high-energy absorption band is a complex transition where we predict, within our SA6-CASSF[8,6] level of theory, contributions from transitions to *S*_2_ up to *S*_5_. As shown in Fig. 2b.

The push–pull effect of TMDCNBD in the *S*_1_ CT state was investigated, and the main results are presented in Section S3 in the SI. To probe the “push” component, methyl groups were added sequentially at positions 1, 5 and 6 of the NBD backbone. The “pull” effect was examined by replacing both cyano substituents with hydrogen atoms. By analysing the magnitude of the charge transfer and the change in the excitation energy (Fig. S6), we observe that simultaneous presence of both electron-donating methyl groups and electron-withdrawing cyano groups is crucial for enhancing the charge-transfer character, accompanied by a decrease in excitation energy. This highlights the importance of the push–pull effect in TMDCNBD.

### 2.2. Aggregation and excitonic effects in the crystal

We analysed the possible aggregation patterns within the crystal packing of TMDCNBD by characterising all dimers found in the crystal within a radius of 8 Å from the central molecule. For this, we used the tools implemented in fromage.^78^ We identified ten different dimers (their structures are presented in Fig. S7 and the cartesian coordinates of all the dimers included in the SI). We also evaluated the exciton couplings for the first excitonic pair (*J*_*s*_1_*s*_2__) of each dimer using the half-energy splitting method. We found values of *J*_*s*_1_*s*_2__ ranging from 0.55 to 61.10 meV (see Table S2 from SI).

Upon photoabsorption, the excitation may delocalise across the different types of aggregates present in the crystal lattice, triggering exciton transport. In certain cases, this process can compete with radiative and nonradiative decay pathways such as internal conversion.^42^ The regime of transport (coherent or incoherent) is determined mainly by the competition between the electronic coupling between the nearest molecular neighbours in the crystal packing *J*_*s*_1_*s*_2__ and the reorganisation energy *λ*. *λ* is associated with the energetic cost of geometry relaxation in the electronic transition and it is an approximated manner to characterise the electron-phonon coupling.^42^ In the case of TMDCNBD, we obtained a value of *λ*_s_1__ = 1.58 eV (See section 4 for details on how *λ* is determined), which is significantly much higher than any of the *J*_*s*_1_*s*_2__ calculated for the ten dimers present in the crystal packing. The weak excitonic coupling compared to *λ* indicates that the excitation will rapidly localise in one TMDCNBD chromophore during the photodynamics, precluding significant excitonic effects, and if transport were to occur, would be *via* incoherent hopping. Additionally, a natural transition orbital analysis of the first excitonic pair, performed on the ground-state optimised dimer structures, shows that for most cases the excitation is localised on a single chromophore (see Fig. S8–S17 in the SI).

**Fig. 3:**
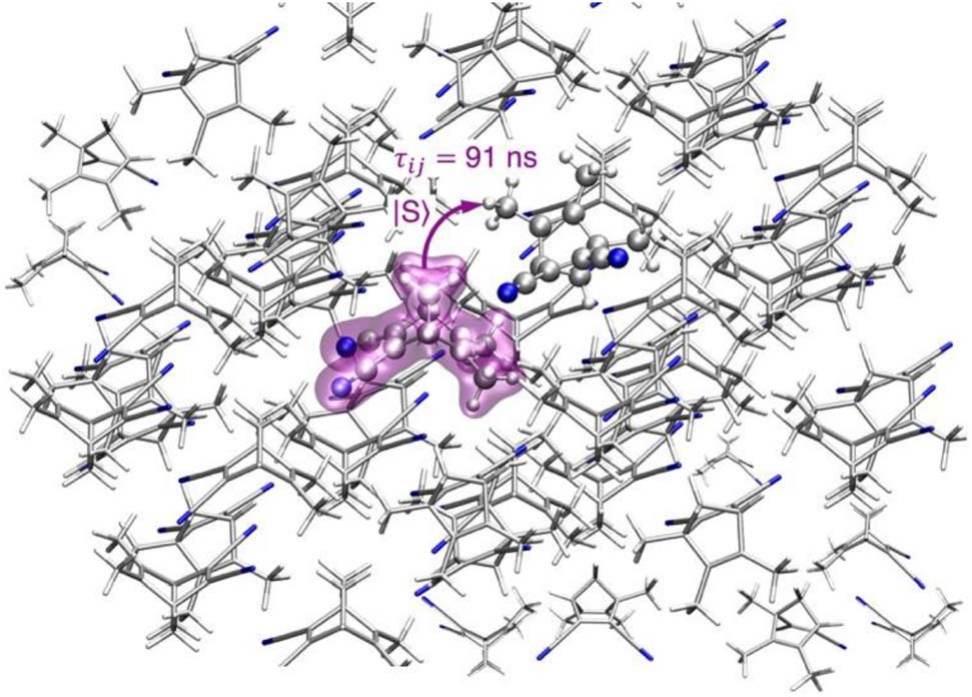
Depiction of exciton transfer for the dimer with highest exciton coupling *J*_*s*_1_*s*_2__ = 61.10 meV in the first pair of states *S*_1_ and *S*_2_. The highlighted molecule represents the position of the excitation about to transfer *via* an incoherent hopping mechanism. The exciton transfer rate constant obtained for this dimer using a Marcus model (see Section 4 for details) is *k*_*ij*_ = 1.1 × 10^7^ s^−1^ giving a transfer time *τ*_*ij*_ = 91 ns.

Considering the dimer with highest electronic coupling 
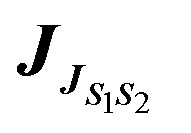
= 61.10 meV (depicted in Fig. 3 within its crystalline environment), we evaluated the exciton transfer rate constant between the molecule *i* and the molecule *j k*_*ij*_, using the Marcus model detailed in Section 4 (eqn (3) and (4)). The predicted exciton rate constant is *k*_*ij*_ = 1.1 × 10^7^ s^−1^ giving an exciton hopping time constant *τ*_*ij*_ = 1/ *k*_*ij*_ = 91 ns. To provide an example of the hopping rates typically observed in systems where transport is important, organic semiconductors exhibit values on the order of 540–580 fs for pure anthracene^105^ and 105–588 fs for carbazole crystals,^106^ obtained using methodologies similar to those employed in this work. These timescales are several orders of magnitude shorter than those obtained for the present system. For example, while the larger exciton couplings in TMDCNBD are on the order of 61 meV and tend to be larger than those in carbazole (23 meV), the smaller rates in TMDCNBD are consistent with its significantly larger reorganisation energy (1.58 eV), in contrast to carbazole, for which the reorganisation energy is around 0.25 eV. The two dimers with the strongest exciton couplings in TMDCNBD (61 and 45 meV for dimers 1 and 2, respectively) exhibit C–H(CH_3_)⋯π(C=C) and C–H⋯N/C(CN) interactions at intermolecular distances on the order of 2.7–3.0 Å. The π–π stacking interactions between the C=C groups are less effective due to the significant displacement from a parallel configuration, resulting in C⋯C distances of 4.3 Å in dimer 2 and 4.8 Å in dimer 5. In contrast, π–π stacking interactions involving the C–N groups occur at shorter distances of 3.6 and 4.2 Å. These intermolecular interactions influence the reorganisation energies and Huang–Rhys factors compared with those obtained in the gas phase.

As we show in Section 2.3, *τ*_*ij*_ is orders of magnitude longer than the nonradiative deactivation simulated *via* NAMD, thus predicting that exciton transport is not a competitive pathway in the TMDCNBD crystal. Additionally, the localisation of the electronic excitation after photon absorption (*J*_*s*_1_*s*_2__ << *λ*_*s*_1__) in combination to the much slower exciton transfer process compared to nonradiative deactivation, shows that the multiscale molecule centred model used in this work is adequate for the description of the excited state dynamics of TMDCNBD in the crystalline phase.

### 2.3. Excited state dynamics

**Fig. 4:**
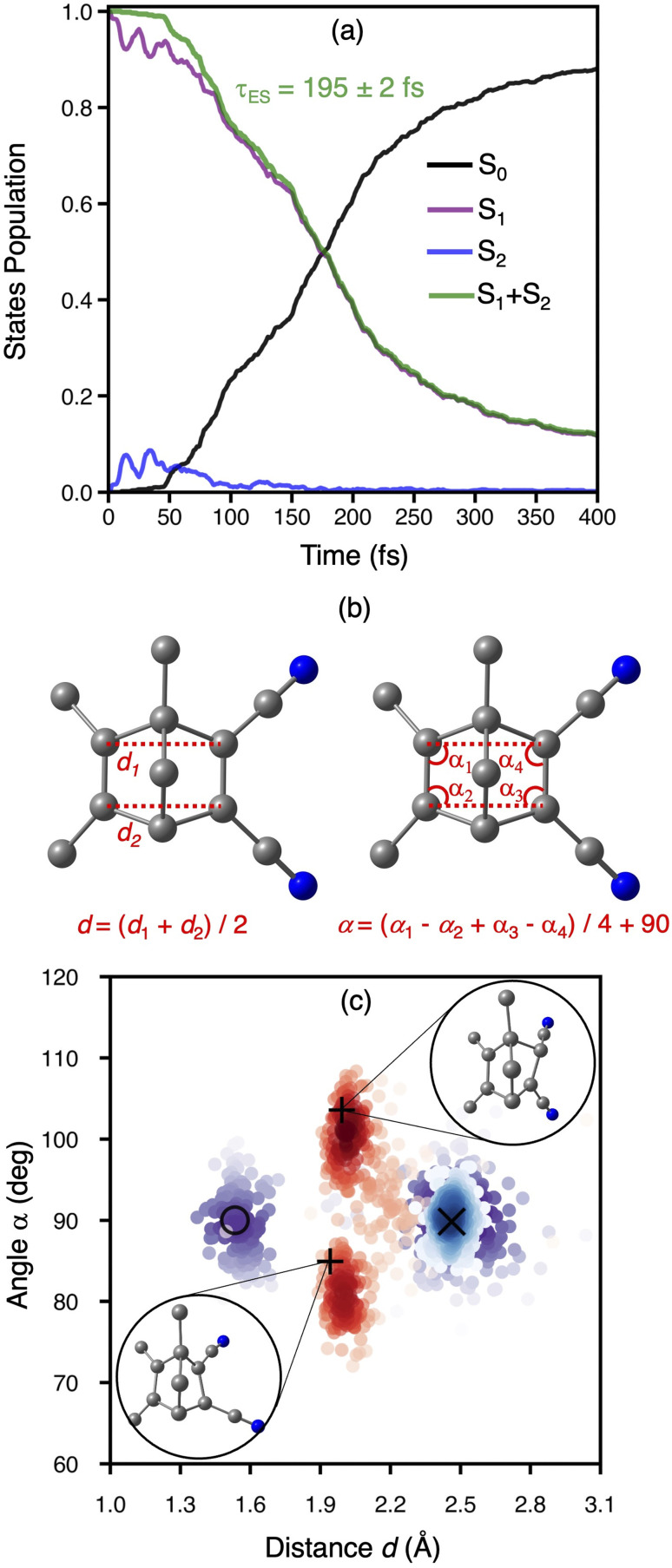
(a) State populations of 520 TMDCNBD trajectories in the molecular crystal. (b) Definition of the average distance between the π_C–C_ bonds, *d*, and the rhomboidal angle among the bond-forming carbon, *α* in TMDCNBD. Hydrogen atoms are omitted for clarity. (c) *d* and *α* distributions of the 520 trajectories for the *S*_1_-FC Wigner-sampled geometries (blue), and the 458 trajectories forming the TMDCNBD and TMDCQC photoproduct distribution after deactivation to the ground state (violet), and the corresponding surface hopping geometries (red). The intensity of the colours represents the accumulation of the points from low to high, evaluated by Gaussian kernel density estimation. The “X”, “O” and “+” symbols mark the positions of the optimised ground state minimum of TMDCNBD, TMDCQC and *S*_1_/*S*_0_-MECI geometries. The geometries of the two *S*_1_/*S*_0_-MECIs are shown in the insets without displaying the hydrogens for a better clarity.

We propagated 520 NAMD trajectories initiated from the Franck-Condon region of the *S*_1_ state (*S*_1_-FC) for 400 fs. The excitation to the *S*_1_-FC region was done using an instantaneous vertical excitation approach.^98^ Thus, no laser effect *via* energy windowing and transition dipole moments were accounted for the initial conditions of the dynamics. With this set of initial conditions, 88% of trajectories reach the ground state after 400 fs, 11.8% and 0.2% of the trajectories remain in *S*_1_ and *S*_2_, respectively. Fig. 4a shows the average state populations in the 400 fs simulation. Within the first 50 fs of dynamics, *S*_1_ transfers a small percentage of population (<10%) to *S*_2_, which is transferred back to *S*_1_ within the following 100 fs. Considering the ultrafast *S*_1_ → *S*_2_ → *S*_1_ population transfer, we obtain the excited-state population from the total excited-state occupation (*S*_1_ + *S*_2_) as shown in Fig. 4a. The excited-state lifetime was therefore obtained following a similar approach to that described in ref. 107. We propose fitting the decay of the total excited-state population fraction using the following function:1
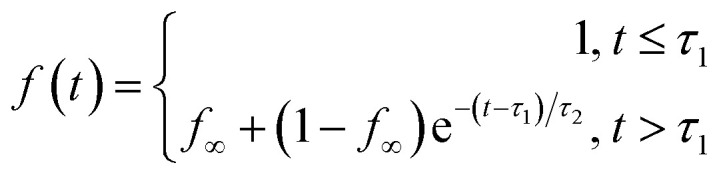
where *τ*_1_ is the latency time to initiate the internal conversion, *τ*_2_ is the exponential decay constant, and *f*_∞_ is the fraction of population that has not decayed within this time constant. The excited-state lifetime (*τ*_ES_) is then given by *τ*_ES_ = *τ*_1_ + *τ*_2_. The calculated excited-state lifetime of TMDCNBD in the crystal is *τ*_ES_ = 195 ± 2 fs at the 95% confidence level. All the fitting parameters are provided in Table S3 in the SI. The lifetime in the crystal is comparable to that predicted for the analogous molecule, DMDCNBD in the gas phase (*τ* = 190 fs).^26^ These results suggest that the environment does not exert a significative effect on the nonradiative deactivation of TMDCNBD towards its photoproducts.

We analysed the excited-state dynamics by projecting the molecular geometries onto two reaction coordinates, the rhomboidal angle among the bond-forming carbon, *α*, and the average distance between the π_C–C_ bonds, *d* as defined in Fig. 4b. Overall, at the beginning of the dynamics (*t* = 0), a narrow 2D-Gaussian-like distribution of *d* and *α* is observed, as expected from Wigner-sampled structures (Fig. 4c).^99^ Over the 400 fs of dynamics, deactivation occurs towards the formation of TMDCQC and TMDCNBD as the main photoproducts. The TMDCNBD and TMDCQC points are accumulated at *d* = 2.46 Å and *α* = 89.4°, and *d* = 1.53 Å and *α* = 90.3°, respectively. Other points are accumulated at intermediate values of *d* that are between the values for TMDCNBD and TMDCQC or even higher values of *d* and *α*, suggesting the formation of other photoproducts (see Section 2.4 for details). The photoreaction proceeds *via* excited-state nonradiative deactivation, passing through two main *S*_1_/*S*_0_ surface-hopping regions, localised around *d* = 2.0 Å and *α* = 81°, and *α* = 101°, respectively (Fig. 4c). An energy distribution analysis shows that most of the surface hops occur at energies below 0.25 eV (Fig. S18 in the SI) suggesting the hops must occur near the crossing seam between *S*_1_ and *S*_0_. The two surface-hopping regions are in fact close to two *S*_1_/*S*_0_ minimal energy conical intersections (*S*_1_/*S*_0_-MECI) with *d* = 1.94 Å and *α* = 84.94°, and *d* = 1.99 Å and *α* = 103.58°, respectively. The symmetric angular distribution found for the *S*_1_/*S*_0_ hopping points indicates two equivalent *S*_1_/*S*_0_ crossing seams around the *S*_1_/*S*_0_-MECIs. Both optimised *S*_1_/*S*_0_-MECIs, within the multiscale OEC model, are isoenergetic and below the energy of the *S*_1_-FC region, explaining why the nonradiative deactivation is ultrafast. A detailed analysis of the structural differences of both MECIs and the interpolated pathways connecting the FC region with the MECIs is included in Section S5 in the SI. A photoproducts distribution analysis reveals that the region of the crossing seam explored during the nonradiative deactivation process does not exert any significant difference on the formation of the photoproducts (see Section S5 for details).

Energetically accessible *S*_1_/*S*_0_-MECIs are lower in energy than the FC region and have been previously reported for NBD systems studied in the gas phase.^24,26–29,96^ Our simulations indicate that the *S*_1_/*S*_0_-MECI is also accessible in the solid state; thus, the photochemistry should be efficient and the system is unlikely to display fluorescence. The competition between photoisomerisation and fluorescence, and the active role played by the surrounding environment, have been demonstrated experimentally for other NBD derivatives in solution,^108–112^ In addition to intrinsic molecular factors, fluorescence is favoured in conditions of polarity and viscosity for which photoisomerisation is disfavoured.

The accessibility to *S*_1_/*S*_0_-MECI contrasts with solids where the crystal provides a more restrictive environment. This was explained by the restricted access to conical intersections (RACI)^113^ model that has been successfully used to address phenomena such as the increase of emission quantum yields of systems in the solid state that are not emissive in solution. In these cases, the environment imposes a steric restriction and the *S*_1_/*S*_0_-MECI region is energetically destabilised above the FC region when going from solution to the solid.^113,114^ However, our studies of the potential energy surface and NAMD suggest that the crystalline environment of TMDCNBD provides sufficient spatial flexibility for the nonradiative decay of NBD in the crystal to allow an energetically accessible *S*_1_/*S*_0_-MECI, consistent with the volumetric analysis discussed in Section 2.1 and Section S1 in the SI (Fig. S1), which indicates that each molecule has a significative space to freely move.

### 2.4. Photoreaction mechanisms in crystalline TMDCNBD and the effect of the environment

**Fig. 5:**
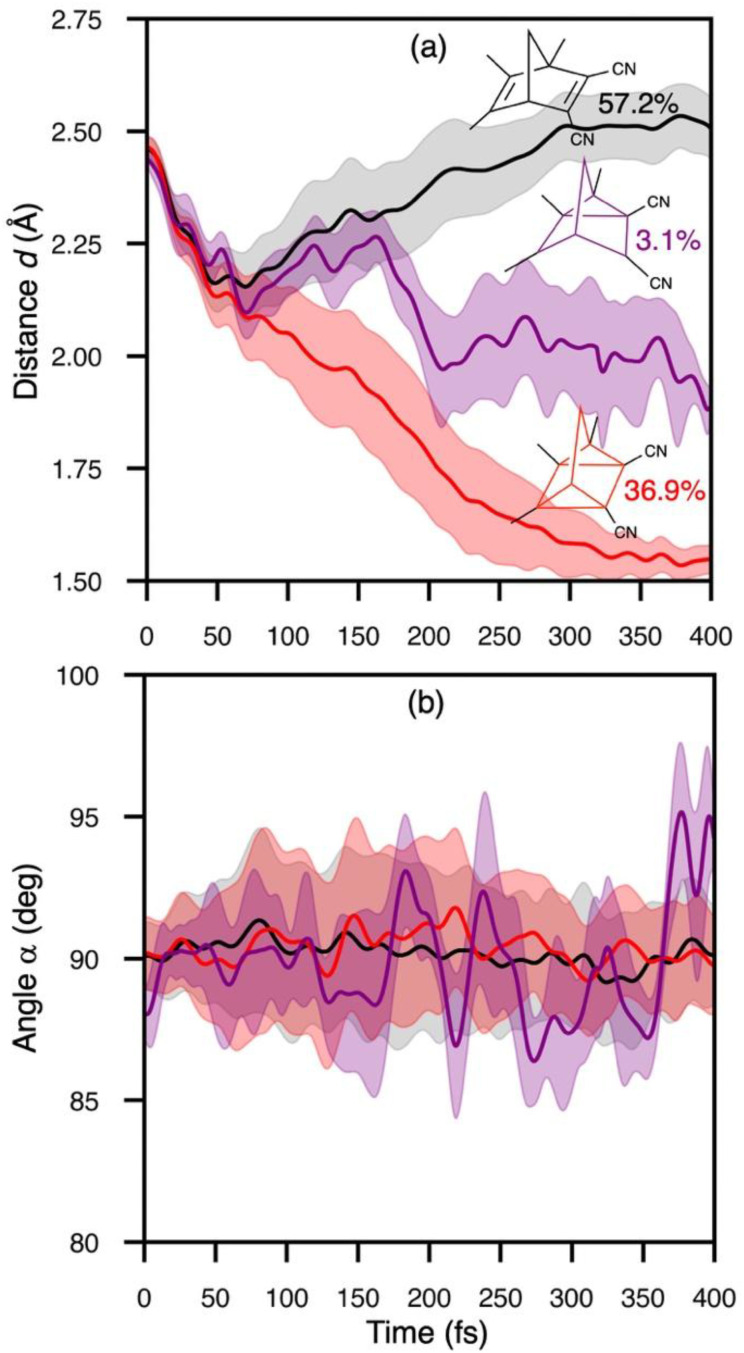
Evolution of the average distance between the π_C–C_ bonds, *d* (a), and the rhomboidal angle among the bond-forming carbon, *α* (b) in TMDCNBD. The insets in panel (a) show the %QY for the formation of the main photoproducts.

We traced the temporal evolution of the π_C–C_-distance *d* and rhomboidal angle *α* to understand the mechanism of the [2 + 2]-photocycloaddition of crystalline TMDCNBD (Fig. 5). The trajectories start from *d* = 2.46 Å and *α* = 89.4°, on average. Within the first 40 fs, *d* substantially decreases below 2.2 Å (Fig. 5a) and *α* oscillates between 85 and 95° (Fig. 5b). From 40 fs onwards, the trajectories diverge in three major pathways towards TMDCNBD, TMDCQC, and a singlet biradical (BR) TMDCBR intermediate. Our trajectories predict a QY of TMDCNBD, TMDCQC and TMDCBR of 57%, 37% and 3%, respectively. The predicted QC : NBD formation ratio for TMDCNBD in the crystal is therefore 64%.

Although TMDCNBD is still the major photoproduct after nonradiative deactivation, the formation QY of the metastable TMDCQC is considerably high. A thermodynamics analysis based on harmonic normal modes calculations of TMDCNBD and TMDCQC in the crystal gives a ΔH^0^_QC−NBD_ = 64.6 kJ mol^−1^, which gives a predicted energy density storage ESD = 0.36 MJ kg^−1^. The predicted ESD is above the minimum acceptable ESD = 0.3 MJ kg^−1^ frequently quoted.^115^ For this analysis, normal modes were obtained at the ωB97X-D/aug-cc-pVDZ level using the structures optimised at the multiscale ωB97X-D/aug-cc-pVDZ/GFN2-xTB level.

**Fig. 6:**
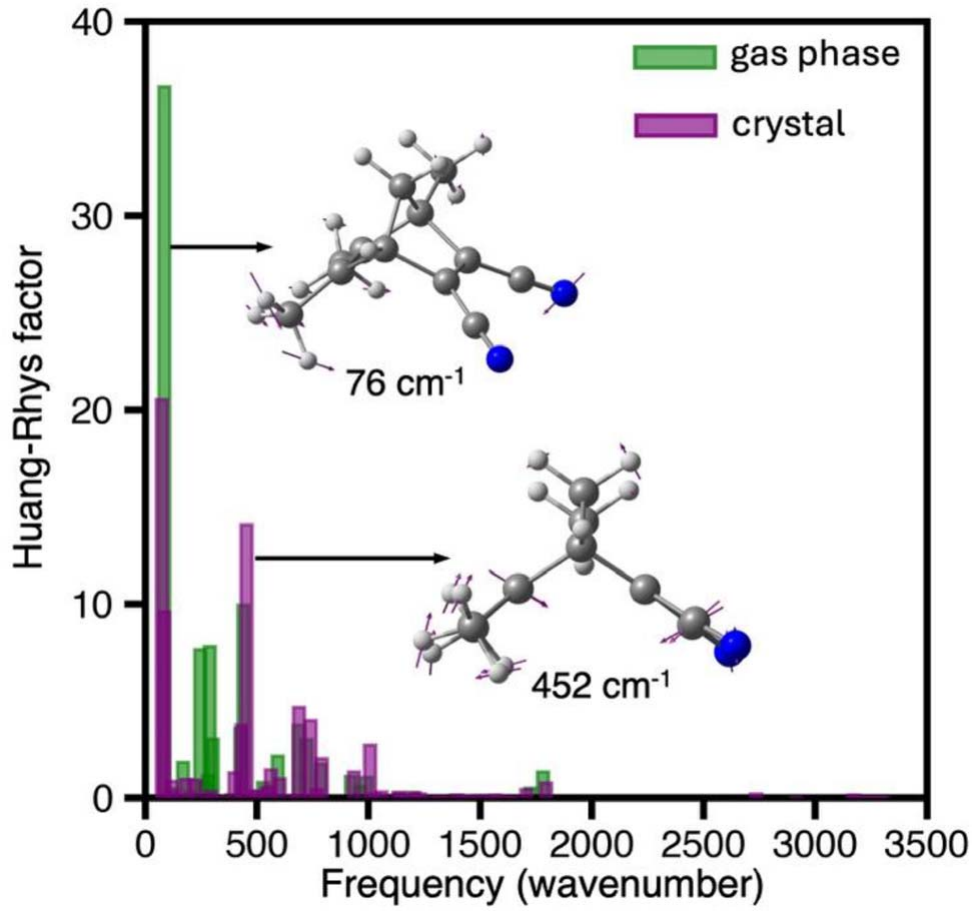
Huang–Rhys (HR) factor for the absorption process to the *S*_1_ state calculated for TMDCNBD optimised in gas phase (green) and optimised in the crystal and embedded in point charges (purple), both obtained at the SA6-CASSCF[8,6]/ANO-S-VPDZ level of theory. The two normal modes showing the highest HR factors in the crystal are depicted in the inset. The calculated frequency of these normal modes are 76 cm^−1^ and 452 cm^−1^, respectively.

To get deeper understanding on the mechanistic origin of the ultrafast dynamics and the substantial structural changes following photon absorption, we calculated the Huang–Rhys (HR) factors for the bright *S*_0_ → *S*_1_ transition of TMDCNBD optimised both in the crystal and in the gas phase and plotted them *vs.* the corresponding normal modes frequencies in *S*_0_ state (Fig. 6). A high Huang–Rhys factor indicates a strong contribution of the corresponding normal mode to the absorption transition, *i.e.* the fraction of the electronic energy going to that specific mode, which can therefore be associated with its “activation” during the excitation. The modes with the highest HR factors in the crystal corresponds to the ring closure motion with a calculated frequency at the SA(6)-CASSCF[8,6]/ANO-S-VPDZ level of 452 cm^−1^ and a mode that primarily involves distortions of the methyl and cyano group with a predicted frequency of 76 cm^−1^ (Fig. 5). The activation of the ring closure mode predicts a big change in the π_C–C_-distance *d* suggesting that the [2 + 2]-photocycloaddition is impulsively light-triggered, whereas activation of the methyl and cyano groups may facilitate the dynamics towards the croasing seam region.

The photoactivation of the 452 cm^−1^ normal mode, with a time period *τ* ≈ 74 fs, explains the substantial drop of *d* observed in the first 50 fs of dynamics. This mode is less active in the gas phase, highlighting the crystalline environment has no restrictive role in the activation of this intramolecular vibration and therefore allowing the ultrafast deactivation.

Taken together, the high ESD, the substantial %QY for formation of the metastable TMDCQC on an ultrafast timescale, and the lowest-energy absorption band centred at *λ*_max_ = 376 nm (3.3 eV; Fig. S2), predicted at the LR-TDDFT/ωB97X-D/aug-cc-pVDZ level of theory and matching with the solar spectrum at the Earth's surface,^30^ indicate that crystalline TMDCNBD is a strong candidate for efficient MOST applications.

## 3. Conclusions

We have employed a fully atomistic multiscale QM/QM′ NAMD framework to elucidate the photodynamics of crystalline TMDCNBD, a push–pull norbornadiene derivative promising for MOST applications.

Our simulations reveal that the crystalline environment preserves the ground-state geometry predicted in the gas phase and affords sufficient free volume for large-amplitude intramolecular motions. Weak excitonic couplings relative to the reorganisation energy led to localised excitations and negligible exciton transport on the sub-nanosecond timescale. Thus, ultrafast nonradiative decay proceeds *via* energetically favourable *S*_1_/*S*_0_ conical intersections enabling efficient [2 + 2]-photocycloaddition.

Although the photoproducts distribution favours TMDCNBD (57%), a significantly high quantum yield is predicted for the metastable TMDCQC (37%), which can store 0.36 MJ kg^−1^, above the practical threshold for MOST systems. A Huang–Rhys factors analysis identifies the photoactivation of a 452 cm^−1^ ring-closure vibration as the dominant photoactive mode, impulsively triggering the reaction coordinate and thus the ultrafast nonradiative deactivation. Overall, this type of push–pull norbornadiene crystalline derivative emerges as a highly promising MOST material, combining ultrarapid photoconversion with a high quantum yield for the formation of the metastable, energy-rich quadricyclane isomer.

Finally, beyond this specific system, our study presents a transferable computational methodology and protocol, and a ready-to-use set of tools for probing ultrafast photochemistry in molecular crystals. Our workflow starts with the use of periodic boundary conditions for the refinement of crystalline structures, followed by the investigation of possible dimeric excitonic pathways to determine whether localised approaches are effective. If the reorganisation energies are larger than the exciton couplings, localised embedding approaches can be applied to investigate the photochemical processes. Subsequently, we use QM:QM′ embedding methods to explore potential energy surfaces and to study the coupling between electronic structure and vibrations through the analysis of Huang–Rhys factors and nonadiabatic dynamics. One of the advantages of this approach is that it enables the use of high-level *ab initio* calculations, including multiconfigurational methods, for the investigation of excited-state dynamics.

## 4. Methods

Our approach combines a multiscale QM/QM′ electrostatic embedding scheme with NAMD propagated with FSSH algorithm thanks to a newly integrated interface between fromage (FRamewOrk for Molecular AGgregate Excitations)^78–80^ and PyRAI^2^MD (Python Rapid Artificial Intelligence Ab Initio Molecular Dynamics).^62,81,82^ fromage implements the Our own N-layered Integrated molecular Orbital and Molecular mechanics (ONIOM)^116^ approach with electrostatic embedding and it is interfaced with various quantum chemical programs to compute excitation energies and hybrid gradients, and supports a variety of molecular or periodic QM methods to compute atomic charges for electrostatic embedding. For instance, the electrostatic potential fitted in the Merz–Singh–Kollman (MK) scheme,^117,118^ restrained electrostatic potential (RESP),^119^ Mulliken population, natural charges,^120^ Hirshfeld charges,^121^ and atoms-in-molecules (AIM) analysis.^78^ and self-consistent Ewald periodic embedding methods in both ground and excited states.^78^ Our implementation enables the use of any of the approaches implemented in fromage in conjunction with PyRAI^2^MD.^122^

**Fig. 7:**
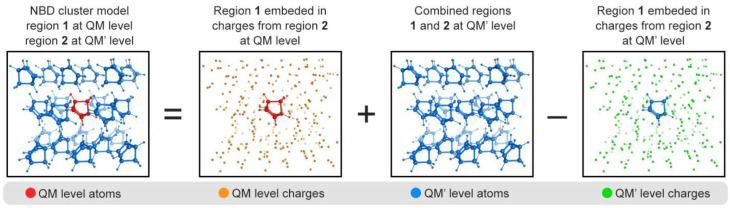
Schematic representation of the two-level ONIOM(QM/QM′) model used in this work.

Photochemical reactions in molecular crystals are generally localized to a single chromophore or chromophore dimer. The induced structural changes are relevant to the chromophore conformation and corresponding intermolecular interactions. This phenomenon suggests the excited-state calculations of a full unit cell under periodic conditions are less pertinent than simplified computations using finite-sized molecular cluster models. Herein, we consider a two-level ONIOM(QM/QM′) scheme with electrostatic embedding to compute the ground- and excited-state potential energy surfaces (PESs, Fig. 7).

The excited-state energy of the molecular crystal using the ONIOM embedded cluster is given by:

2 *E*_QM/QM′_(1∪2) = *E*^PCE^_QM_(1) + *E*_QM′_(1∪2) − *E*^PCE^_QM′_(1)

*E*^PCE^_QM_(1) is the excited-state energy of the central region 1 shown in Fig. 7, computed with the QM method of higher hierarchy. The centrally excited molecule is embedded in the point charges from region 2. The point charge embedding (PCE) accounts for the Coulomb interaction between the excited state region 1 and the ground state region 2. *E*_QM′_(1∪2) is the total ground state energy of the regions 1 and 2 combined using the QM′ method. *E*^PCE^_QM′_(1) is the ground-state energy of region 1 at the QM′ level considering point charges. Subtracting this term from *E*_QM′_(1∪2) approximately removes the double-counted Coulomb interactions between regions 1 and 2. The last two terms provide essential short-range non-Coulomb interactions in the ground state producing a reliable PES. The gradients are computed accordingly. Because the non-Coulomb interactions are evaluated in the ground state, the energetic contributions are equivalent in all electronic states in *E*^PCE^_QM_(1). We restricted the calculation of the nonadiabatic couplings (NACs) to region 1, setting the NACs components of the atoms in region 2 to zero. This approach has been used before for multiscale QM/MM NAMD.^123^ Thus, the nonadiabatic hopping probability is governed only by the region described at the multiconfigurational level. With this, we also prevent an unphysical drain of kinetic energy to the environment by distributing the excess of kinetic energy at the time of the hopping only to the atoms in region 1.

We started from the experimental crystalline structure of TMDCNBD (CCDC 1118944),^124^ followed by a relaxation of the structure using periodic-DFT with the PBE-D2 functional with plane wave cut-off of 60 Ry and ensuring Monkhorst–Pack *k*-point convergence (1 × 2 × 2). Quantum Espresso v6.3 (ref. 125 and 126) software package was used for this part. We then applied the ONIOM embedded cluster model considering a single photoexcited TMDCNBD molecule in the region 1. The environmental region 2, comprising 79 TMDCNBD molecules, was generated applying a spherical cutoff of 12 Å from the centre of region 1 for the dynamics simulations and optimisations when only one TMDCNNBD chromophore is included in region 1. Normal modes of the optimised structure were computed considering ad-hoc point charges to represent the electrostatic environment. For dimers optimisation, starting from a 2 × 2 × 2 supercell, a spherical cutoff of 8 Å was applied generating a region 2 comprising 47 TMDCNBD molecules.

**Fig. 8:**
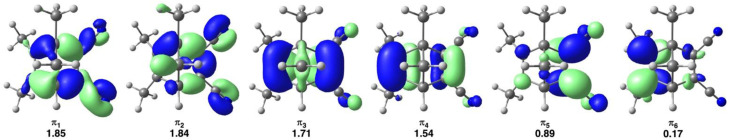
Illustration of the [8,6] active space of TMDCNBD in the crystal along with the occupations averaged over six electronic states, calculated with SA6-CASSCF[8,6]/ANO-S-VDZP.

The multiscale electronic structure calculations employ the CASSCF method as implemented in Open Molcas v19.11,^127^ to describe the QM region 1 and generate correlated excited-state PESs. Based on our previous study of the photodynamics of DMDCNBD derivative in the gas phase,^26^ we considered a [8,6] active space computed with 6 states averaged (SA6) along with the ANO-S-VDZP basis set.^128^ The active space comprises the 4 π-electrons, 4 π-orbitals involved in bond formations and additional four π-electrons and two π-orbitals of the cyano groups, forming an [8,6] active space as shown in Fig. 8. The selection of the π-orbitals was based on the LR-TDDFT results obtained with the range-separated ωB97X-D3 functional with the aug-cc-pVDZ basis set as implemented in Gaussian 16 RevA.03.^129^

The QM-charges were fitted from the electrostatic potential in the MK scheme with HF/ANO-S-VDZP. This method produces compatible charges for the CASSCF calculations in the NAMD simulations. The QM′-charges were computed by the semiempirical extended tight-binding GFN2-xTB method as implemented in the xTB v 5.5.1 software package,^130^ which generates consistent partial charges for the QM′-calculations for the region 1. GFN2-xTB was also used to account for the energetic contributions from the crystal environment. For the optimisations at the (LR-TD)DFT/ωB97X-D/aug-cc-pVDZ/GFN2-xTB level of theory, the embedding of region 1 at the high level was done using the RESP scheme, instead.

1000 nuclear geometries of TMDCNBD in the crystal were sampled using a harmonic Wigner distribution at the zero-point energy level considering the harmonic vibrational normal modes of TMDCNBD in region 1, calculated at the ωB97X-D/auc-cc-pVDZ level, embedded in RESP charges from region 2 obtained at the same level of theory. These initial conditions were used to calculate the absorption spectrum shown in Fig. 2 and Fig. S2 using the nuclear ensemble approach with a gaussian broadening factor of 0.05 eV and *T* = 298.15 K. A similar scheme considering 800 nuclear geometries of TMDCNBD in gas phase and embedded within the polarizable continuum model (PCM), within the integral equation formalism,^131^ as implemented in Gaussian 16 Rev A.03 was followed to obtain the absorption spectra in gas phase and acetonitrile, respectively. The Wigner sampling was done with the set of tools available in fromage^122^ for the case of CASSCF and with the Newton-X v 2.6 ^76^ platform for the ωB97X-D calculations. In all cases, the absorption spectra were calculated using an in-house set of codes.

The multiscale NAMD simulations were performed within a frozen crystalline environment and employing the Tully's FSSH original formula based on the product of the time-independent NACs and velocities.^70,71^ The electrostatic response of the environment across different initial conditions and for a selected FSSH trajectory was analysed (see Section S7 for details). Only NACs between adjacent states were considered, assuming zero coupling between nonadjacent states. This approximation accelerates notably the simulations as the calculation of NACs is the most time-consuming part. Phase corrections were applied at every timestep as the NACs phase is arbitrary in CASSCF calculations.^132^ We used the microcanonical ensemble (NVE) with a nuclear integration timestep of 0.5 fs up to a total simulation time of 400 fs. The FSSH calculations integrated the nuclear amplitude with a step size of 0.02 fs (*i.e.*, 25 substeps) and applied an energy-based decoherence correction of 0.1 Hartree to the nuclear amplitude.^133^ At every event of surface hopping, the momenta were rescaled isotropically to ensure energy conservation. Our trajectory analysis removed those trajectories with incorrect state populations, *e. g.*, exceeding 0–1. As a result, we obtained 520 trajectories from the *S*_1_-FC region out of the 600 initially launched.

Due to the problems of energy conservation caused by the instabilities of the CASSCF active space across the dynamics, especially for systems showing a substantial molecular change as TMDCNBD → TMDCQC, a convergence analysis for the excited-state population lifetime and for the photoproducts %QY was performed, checking energy conservation at different times of the dynamics showing the results and conclusions stand in all cases (see section S6 in the SI for details).

The natural transition orbitals obtained at the SA6-CASSCF[8,6] and XMS(6)-CASPT2 levels were calculated using the wavefunction analysis (WFA) libraries as implemented in OpenMolcas v23.10,^134^ processed with Pegamoid to create the cubefiles and visualised with Chemcraft. The natural transition orbitals calculated at the LR-TDDFT/ωB97X-D/aug-cc-pVDZ were obtained with the set of tools implemented in the Gaussian 16 RevA.03 software package.^129^ The Huang–Rhys factor values presented in Fig. 6 were obtained with the FCclasses3 v 3.0.4 (ref. 135)

To study exciton transport within the crystal packing, we considered the dimer model. All the dimers present in the crystal packing were identified using the set of tools implemented in fromage.^79^ We optimised all the dimers within the ONIOM embedded cluster model, considering a spherical cutoff of 8 Å was applied generating a region 2 comprising 47 TMDCNBD molecules. The electronic properties of the dimers were computed considering RESP point-charges electrostatic embedding obtained at the ωB97XD/aug-cc-pVDZ level of theory. We used this approach in previous works to model exciton transport in molecular crystals.^106,136,137^

The exciton hopping rate constants *k*_*ij*_ were computed by using a Marcus-like equation:3
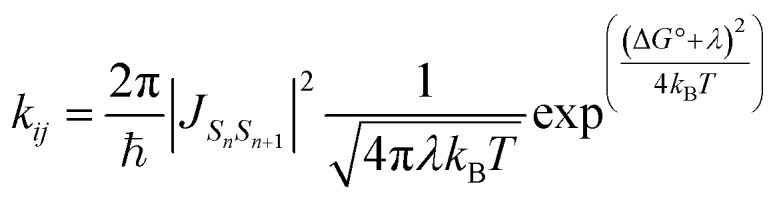
where, 
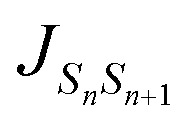
 is the exciton coupling for the pair of excitonic states *S*_*n*_ and *S*_*n*+1_, Δ*G*° is the adiabatic Gibbs free energy associated with the process and *λ* the associated reorganization energy. In our simulations Δ*G*° = 0 as we consider a homodimer where both monomers have identical environments. Thus, the *S*_0_ → *S*_*n*_ excitation occurring in one monomer is exactly counterbalanced by the opposite *S*_*n*_ → *S*_0_ occurring in the other monomer. The reorganisation energy *λ* was calculated as:

4 *λ*_*s*_1__ = *E*_*S*_1__(*R*_min *s*_0__) − *E*_*S*_1__(*R*_min *s*_1__) + *E*_*S*_0__(*R*_min *s*_1__) − *E*_*S*_0__(*R*_min *s*_0__)

Finally, in eqn (3), *T* stands for the absolute temperature and ℏ is the reduced Planck constant. In previous works, we showed that the Marcus model provides a reliable estimate of the timescale of exciton hopping in organic crystals within the incoherent regime, where the electronic coupling is small.^106,136,137^

## Author contributions

FJH: code development, visualisation, conceptualisation, computational work, formal analysis, writing – review & editing original draft. JMC: code development. JL: code development, conceptualisation, review & editing. SAL: visualisation, conceptualisation, review & editing, funding acquisition, project supervision. RCO: visualisation, conceptualisation, writing – review & editing oridinal draft, formal analysis, funding acquisition, project supervision.

## Conflicts of interest

There are no conflicts to declare.

## Supplementary Material

SC-017-D5SC07670F-s001

## Data Availability

Data supporting this publication is obtained from the supplementary information (SI). Supplementary information: contains: volumetric analysis, excited-state and spectroscopic characterisation, push-pull effect analysis, crystal packing and exciton analysis., statistic analysis of excited-state dynamics, potential energy surface characterisation, minimum energy conical intersection geometries characterisation, energy conservation analysis in the nonasiabatic dynamics, product distribution analysis and electrostatic response of the environment. See DOI: https://doi.org/10.1039/d5sc07670f.
