# Supplementary material for: Understanding the photochemistry of a crystalline push–pull norbornadiene photoswitch
Source: Chem Sci. 2026 Feb 10;17(14):7157–70. doi: 10.1039/d5sc07670f (PMC12910361; doi:10.1039/d5sc07670f)
Supplement: SC-017-D5SC07670F-s001 [file SC-017-D5SC07670F-s001.pdf]

Supplementary Information (SI) for Chemical Science.
This journal is © The Royal Society of Chemistry 2026

Electronic Supporting Information:

Understanding the photochemistry of a crystalline push-pull norbornadiene photoswitch

Federico J. Hernández,[a]* Jordan M. Cox,[b] Jingbai Li,[c]* Steven Lopez,[b]* and Rachel Crespo-Otero[d]*

[a]Department of Chemistry, Queen Mary University of London, Mile End Road, London E1 4NS, UK.
[b]Department of Chemistry and Chemical Biology, Northeastern University, Boston, MA 02115, U.S.A.
[c]Hoffmann Institute of Advanced Materials, Shenzhen Polytechnic, 7098 Liuxian Blvd, Nanshan District, Shenzhen, 518055, People's Republic of China.
[d]Department of Chemistry, University College London, London WC1H0AJ, U. K.

Correspondance to: f.hernandez@qmul.ac.uk - lijingbai@szpu.edu.cn – s.lopez@northeastern.edu – r.crespo-otero@ucl.ac.uk

S1. Volumetric analysis of TMDCNBD in the crystal

Using the tools available in *fromage*[1,2] we calculated the volume index V_i which is ratio between the Voronoi and van der Waals (vdW) volumes $V_i = V_{Voronoi} / V_{vdW}$. V_i thus gives an extent of the molecular flexibility and for TMDCNBD optimised in the crystal is $V_i = 1.55$, meaning that TMDCNBD has the 55% of its vdW volume to freely move in the crystal. A graphical representation of the available volume for TMDCNBD to freely move within the molecular crystal is presented in Figure S1.

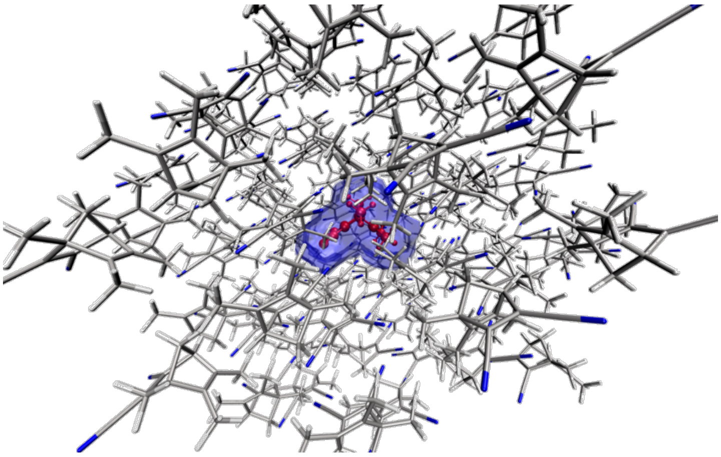

Figure S1. Depiction of the available volume for TMDCNBD to freely move in the molecular crystal.

S2. Excited states characterisation

Table S1: Vertical excitation energies (in eV) with the principal electronic configurations (shown in brackets), and oscillator strengths (f) of TMDCNBD in the crystal and in gas phase, calculated with SA6-CASSCF[8,6]/ANO-S-VDZP, XMS(6)-CASPT2[8,6]/ANO-S-VDZP and with LR-TDDFT/ωB97X-D/aug-cc-PVDZ. The active space is shown in Section 4 of the manuscript.

Level of theory	S_1		S_2		S_3	
			Crystal			
	ΔE	f	ΔE	f	ΔE	f
SA6-CASSCF[8,6]	5.19 (ππ*)	0.005	7.13 (σπ*)	0.012	7.26 (ππ*)	0.278
XMS(6)-CASPT2[8,6]	3.90 (ππ*)	0.004	5.39 (ππ*)	0.330	6.41 (σπ*)	0.006
LR-TDDFT/wB97X-D	3.47 (ππ*)	0.004	4.76 (ππ*)	0.143	5.74 (σπ*)	0.002

			Gas phase			
SA6-CASSCF[8,6]	5.59 ($\pi\pi^*$)	0.009	7.30 ($\sigma\pi^*$)	0.016	7.33 ($\pi\pi^*$)	0.236
XMS(6)-CASPT2[8,6]	4.24 ($\pi\pi^*$)	0.006	5.57 ($\pi\pi^*$)	0.348	6.58 ($\sigma\pi^*$)	0.009
LR-TDDFT/wB97X-D	3.79 ($\pi\pi^*$)	0.007	4.89 ($\pi\pi^*$)	0.145	5.78 ($\sigma\pi^*$)	0.006

The crystalline packing, however, produces a significant red shift in the excitation energies to the lowest three singlet excited states (Table 1). The S_1, S_2, and S_3 excitation energies of TMDCNBD crystal, computed with SA6-CASSCF[8,6]/ANO-S-VDZP, are 0.4, 0.17, and 0.07 eV lower than the gas-phase values, and 0.34, 0.18 and 0.17 eV lower than the gas-phase values when computed with XMS(6)-CASPT2/SA6-CASSCF[8,6]/ANO-S-VDZP. The character of the excited states are the same when going from the gas phase to the crystal. At the SA6-CASSCF[8,6] level, S_1 has a $\pi\pi^*$ character with partial charge transfer (CT) of 0.59 **e** in the crystal and 0.54 **e** in the gas phase from the methyl to the cyano groups. The character of S_2 and S_3 is $\sigma\pi^*$ and $\pi\pi^*$, respectively. However, these states are inverted at the XMS(6)-CASPT2/SA6-CASSCF[8,6]/ANO-S-VDZP level (Table S1 and Figures S3 and S4). The major shift is observed in the transition to S_1 which is likely caused by the higher CT character induced by the electrostatic environment of the crystal. Linear-response time-dependent DFT (LR-TDDFT) calculations using the ωB97X-D[3] functional along with the aug-cc-pVDZ[4] basis set are in very good agreement with these results obtaining a red shift of 0.32, 0.13, 0.04 eV when comparing transitions to S_1, S_2 and S_3, respectively, in the crystal with respect to the gas phase. S_1 state has $\pi\pi^*$ with partial CT of 0.55 **e** in the crystal and 0.50 **e** in gas phase, whereas S_2 and S_3 have a $\sigma\pi^*$ and $\pi\pi^*$ character, in agreement with XMS(6)-CASPT2/SA6-CASSCF[8,6]/ANO-S-VDZP.

In Figure S2 we compare different absorption spectra obtained within the nuclear ensemble approach (NEA), considering 800 Wigner-sampled geometries: (a) computing normal modes and excitations in the gas phase; (b) normal modes in the gas phase and excitations including the electrostatic environment of the cluster via point charges embedding; (c) both normal modes and excitations including the point charges for the structure optimised within the ONIOM embedding cluster approach (OEC); (d) absorption spectra in acetonitrile computed using the polarizable continuum model (PCM) to represent the solvent as implemented in Gaussian 16 Rev C.01 software.[6] This strategy has shown success to reproduce the absorption spectra of complex photoswitches with high level of accuracy.[7] All the spectra were calculated at the LR-TDDFT/ωB97XD/aug-cc-pVDZ level of theory using Gaussian 16 Rev C.01 to compute the energies and Newton-X to compute the NEA.[8] The spectra and the contributions of the lowest five excited states were then calculated using an in-house code, freely available in: https://github.com/federico2099/abs-spectra-tool.git, considering a gaussian broadening factor of 0.075 eV, T = 298.15 K and a refractive index η = 1 except for the case in acetonitrile where the refractive index η = 1.34 of this solvent was used.

Comparing the results obtained for gas phase and gas phase with point charges embedding (Figure S2 panels a and b respectively), it can be seen how the electrostatic environment stabilises the charge transfer state inducing a redshift in the spectrum. Additionally, a decrease in the extinction coefficient is also registered from 385 M^{-1} cm^{-1} in GP to 212 M^{-1} cm^{-1} in gas phase with point charges embedding. A further redshift is obtained when considering the OEC model (Figure S2c) along with an increase in the extinction coefficient from 212 to 262 M^{-1} cm^{-1}. Finally, the electrostatic environment produced by the acetonitrile solvent also stabilises the charge-transfer state producing results in excellent agreement with the experiments in solution. The maximum of the first absorption in our calculations is obtained at 373 nm with an extinction coefficient of 230 M^{-1} cm^{-1}. These results are in excellent agreement with experiments that reported the maximum of the same band at 334 nm with an ε_{max} = 212 M^{-1} cm^{-1}.

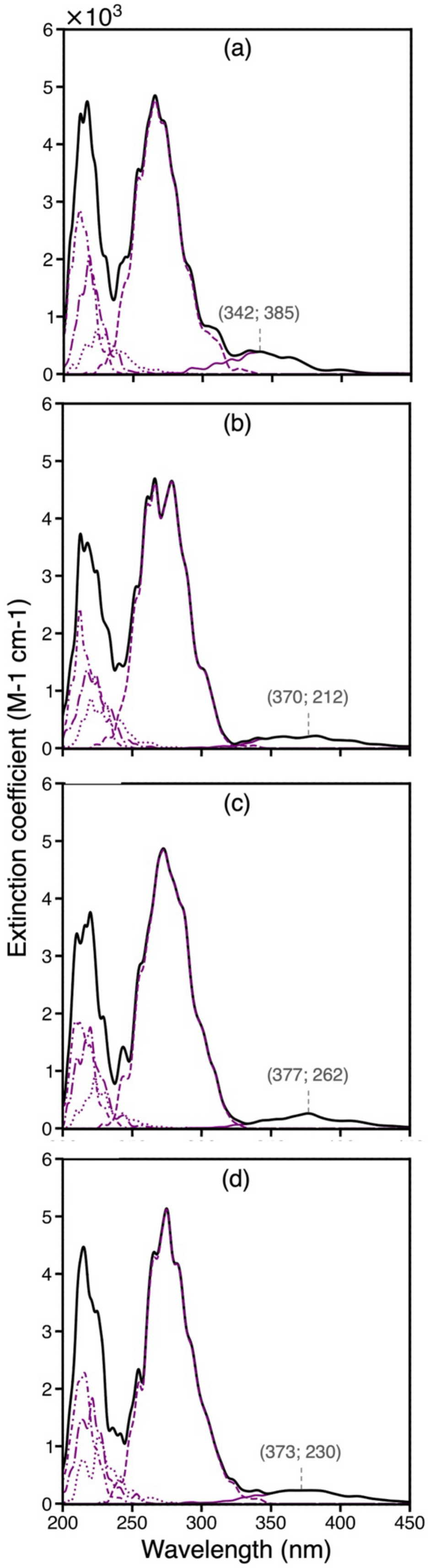

Figure S2: Absorption spectra calculated at the LR-TDDFT/wB97XD/aug-cc-pVDZ level of theory within the nuclear ensemble approach for (a) TMDCNBD in the gas phase, (b) TMDCNBD optimised in the gas phase but excitations computed considering the electrostatic environment produced by a cluster of 79 molecules via point-charges approximation, (c) TMDCNBD optimised in the crystal within the ONIOM

embedded cluster model and (d) TMDNBD optimised in acetonitrile considering the polarizable continuum model (PCM). For each case, considering 800 Wigner sampled-structures. The purple traces show the contributions of the lowest five adiabatic excited states.

The natural transition orbitals (NTOs) obtained at the SA(6)-CASSCF[8,6] and XMS(6)-CASPT2 levels were calculated using the wavefunction analysis (WFA) libraries as implemented in Molcas v23.10[5], processed with Pegamoid to create the cubefiles and visualised with Chemcraft. The NTOs obtained at the LR-TDDFT/wB97X-D3/aug-cc-pVDZ were obtained with the set of tools implemented in the Gaussian 16 RevA.03 software package.[6] Below we show the NTOs calculated for TMDCNBD in the crystal and in gas phase.

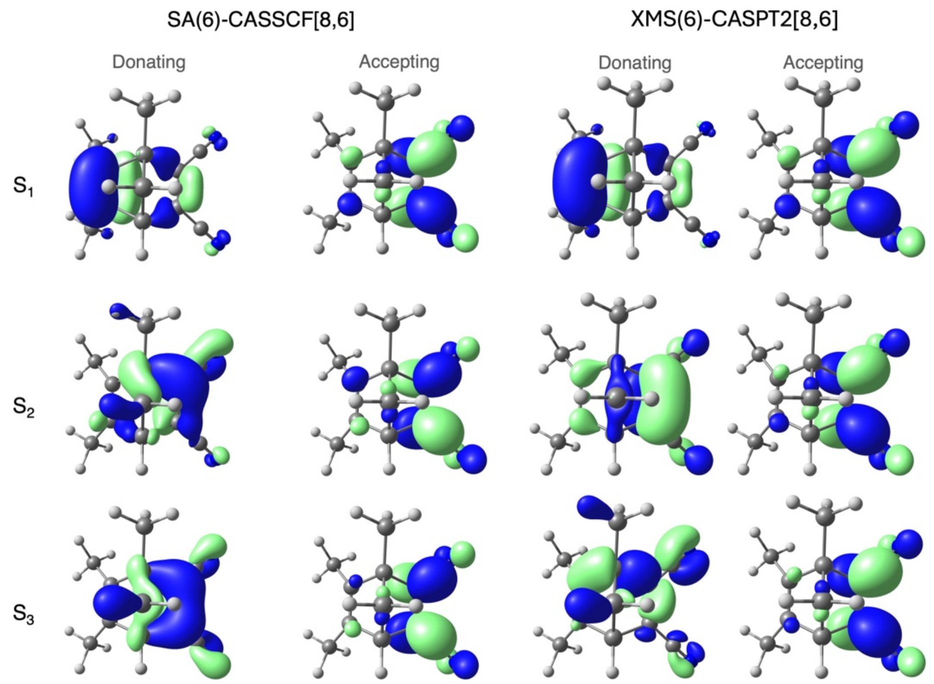

Figure S3. Natural transition orbitals (isovalue = 0.04) of the lowest three transitions of TMDCNBD in the crystal calculated with SA(6)-CASSCF[8,6]/ANO-S-VDZP and with XMS(6)-CASPT2[8,6]//SA(6)-CASSCF[8,6]/ANO-S-VDZP.

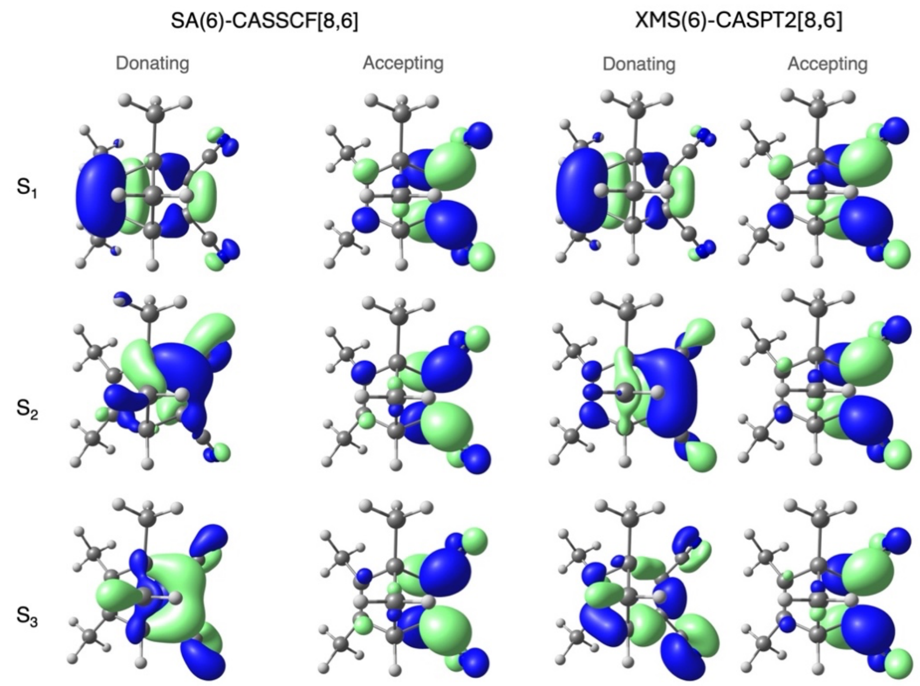

Figure S4. Natural transition orbitals (isovalue = 0.04) of the lowest three transitions of TMDCNBD in gas phase calculated with SA(6)-CASSCF[8,6]/ANO-S-VDZP and with XMS(6)-CASPT2[8,6]//SA(6)-CASSCF[8,6]/ANO-S-VDZP.

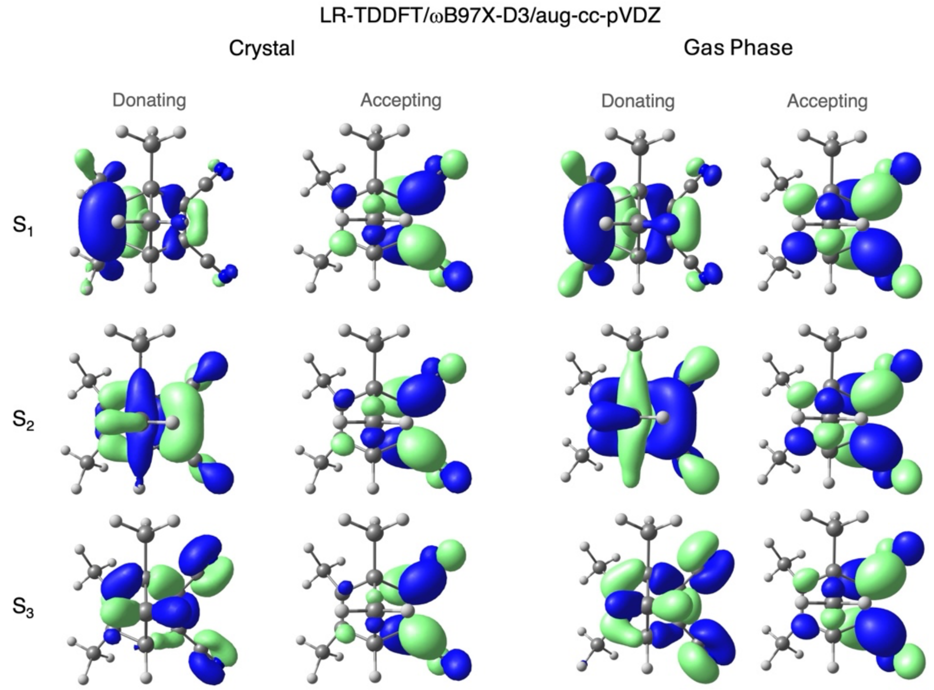

Figure S5. Natural transition orbitals (isovalue = 0.04) of the lowest three transitions of TMDCNBD in the crystal and in gas phase calculated with LR-TDDFT/ωB97X-D3/aug-cc-pVDZ.

S3. Push-pull effect on TMDCNBD

To probe the "push" effect, we considered the sequential addition of one, two and three methyl groups at positions 1, 5 and 6 of the NBD backbone (see Figure S6a for a representation of the molecular structure and the nomenclature of the positions). To examine the "pull" effect, both cyano substituents at positions 2 and 3 were replaced with hydrogens atoms. Overall, we conclude that the simultaneous presence of electron-donating methyl groups and electron-withdrawing cyano groups is crucial for enhancing the charge-transfer character, with a concomitant decrease in the excitation energy. Methyl substitution at positions 5 and 6 leads to a significant increase in the charge-transfer contribution, whereas a methyl at position 1 behaves as a spectator and does not participate in the charge-transfer process.

In the absence of electron-withdrawing groups, a charge transfer of 0.28 **e** is obtained for the NBD derivative methylated at positions 5 and 6. This value is comparable to that obtained when only the electron-withdrawing cyano groups are present at positions 2 and 3, with no methyl substitution, for which a charge transfer of 0.35 **e** is computed. The "pull" effect is particularly evident in the red shift of the excitation energies, with a shift of 0.73 eV relative to the system methylated at positions 1, 5 and 6 (compare systems 5 and 6 in Figure S6c). Nevertheless, it is the combined "push-pull" effect that ultimately produces a pronounced charge-transfer character of 0.51e and a redshift of 1.21 eV with respect to the system containing only methyl substitution, enabling TMDCNBD to absorb in the near-UV region.

Charge-transfer values were obtained from natural population analysis at the DFT/aug-cc-pVDZ and LR-TDDFT/aug-cc-pVDZ levels of theory, using the Gaussian NBO Version 3.1 code,[9] as implemented in the Gaussian 16 (Rev C.01) software package.[6]

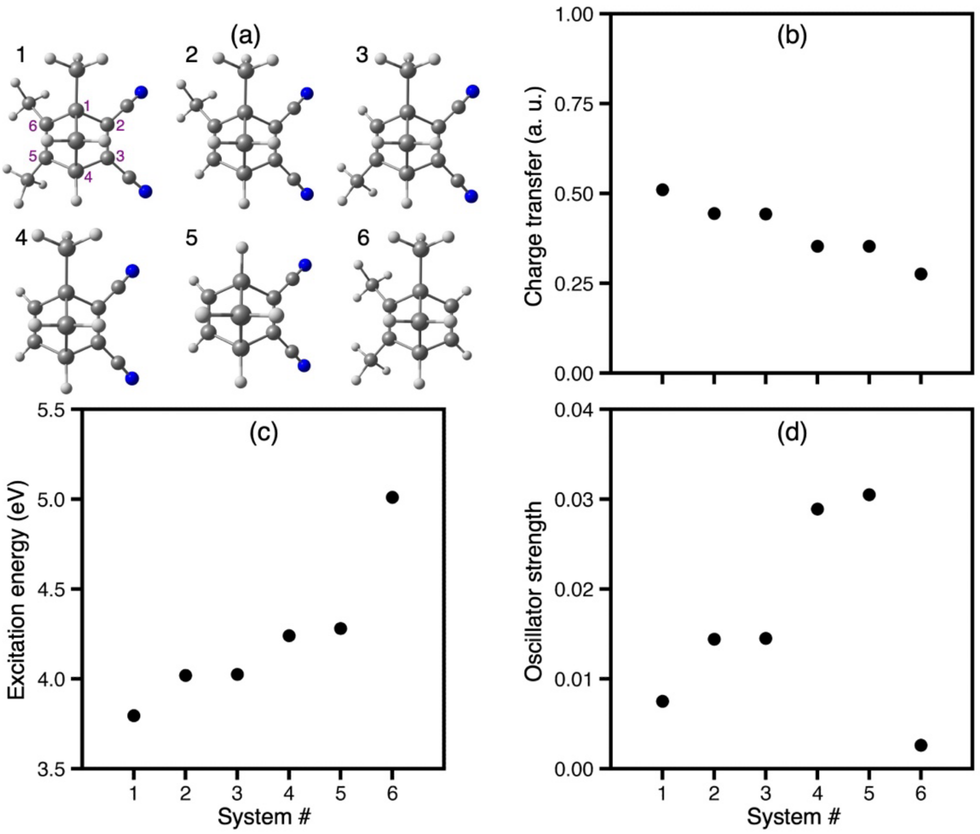

Figure S6. Molecular structures of the norbornadiene derivative studied in this work showing different degrees of substitution with electron-donating methyl groups at positions 1, 5 and 6, and electron-withdrawing cyano groups at positions 2 and 3. The six systems considered are labelled 1–6, and this numbering is used consistently across all panels. The numbers in purple depicted in the structure number 1 represent the substitution position labels. (b) Charge transfer (in atomic units) from the electron-donating to the electron-withdrawing region of the molecule, obtained from natural population analysis for the six systems shown in panel (a). $S_0 \rightarrow S_1$ excitation energies (c) and oscillator strengths (d) obtained for the same systems. The calculations where done with DFT/ωB97XD/aug-cc-pVDZ and LR-TDDFT/ωB97XD/aug-cc-pVDZ in gas phase using Gaussian 16 (Rev C.01).

S4. **Crystal packing and exciton couplings**

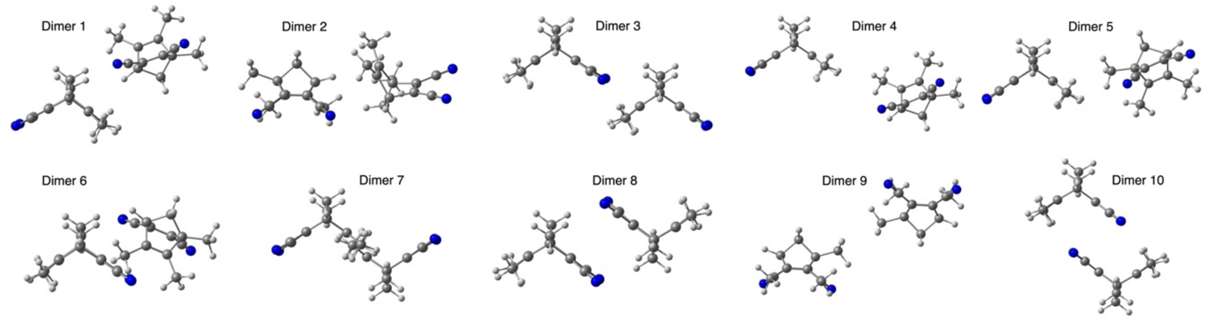

Figure S7. Dimer structures present in the crystal packing and optimised within the multiscale ONIOM embedded cluster model at the ωB97X-D/aug-cc-pvdz/GFN2-xTB level of theory.

Table S2: Vertical excitation energies ΔE (in eV), oscillator strengths (f) and electronic coupling J (in meV) calculated for the lowest two excited states of the ten dimers found in the crystal packing.

Dimer	ΔE_{S_1}	f_{S_1}	ΔE_{S_2}	f_{S_2}	$J_{S_1S_2}$
1	3.4758	0.0043	3.5980	0.0042	61.10
2	3.4550	0.0051	3.5445	0.0046	44.75
3	3.4292	0.0057	3.4751	0.0042	22.95
4	3.4630	0.0038	3.5053	0.0044	21.15
5	3.4762	0.0043	3.5027	0.0056	13.25
6	3.4738	0.0043	3.4995	0.0056	12.85
7	3.4980	0.0013	3.4986	0.0063	3.00
8	3.4419	0.0000	3.4476	0.0049	2.85
9	3.4936	0.0030	3.4947	0.0061	0.55
10	3.4388	0.0044	3.4399	0.0001	0.55

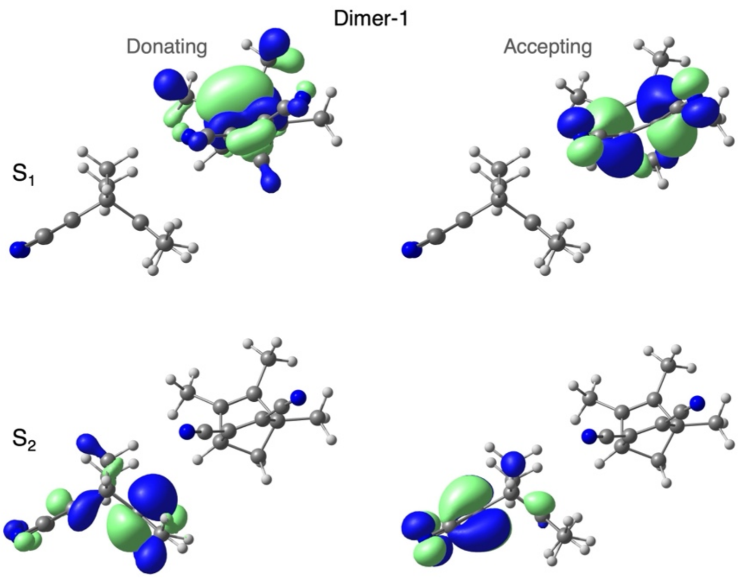

Figure S8. Natural transition orbitals (isovalue = 0.04) of the lowest two transitions of TMDCNBD Dimer-1 in the crystal calculated with LR-TDDFT/ωB97X-D3/aug-cc-pVDZ.

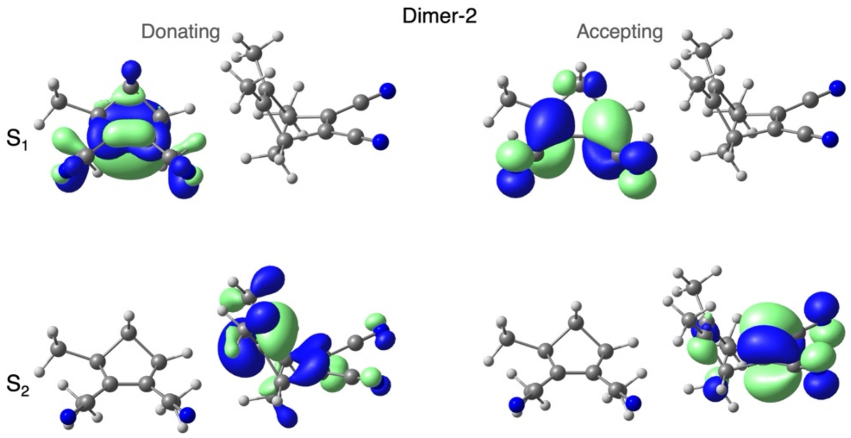

Figure S9. Natural transition orbitals (isovalue = 0.04) of the lowest two transitions of TMDCNBD Dimer-2 in the crystal calculated with LR-TDDFT/ωB97X-D3/aug-cc-pVDZ.

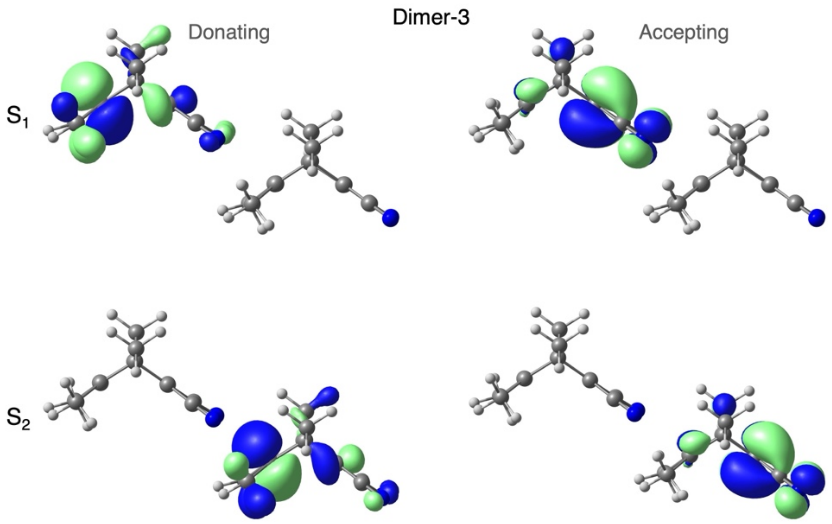

Figure S10. Natural transition orbitals (isovalue = 0.04) of the lowest two transitions of TMDCNBD Dimer-3 in the crystal calculated with LR-TDDFT/ωB97X-D3/aug-cc-pVDZ.

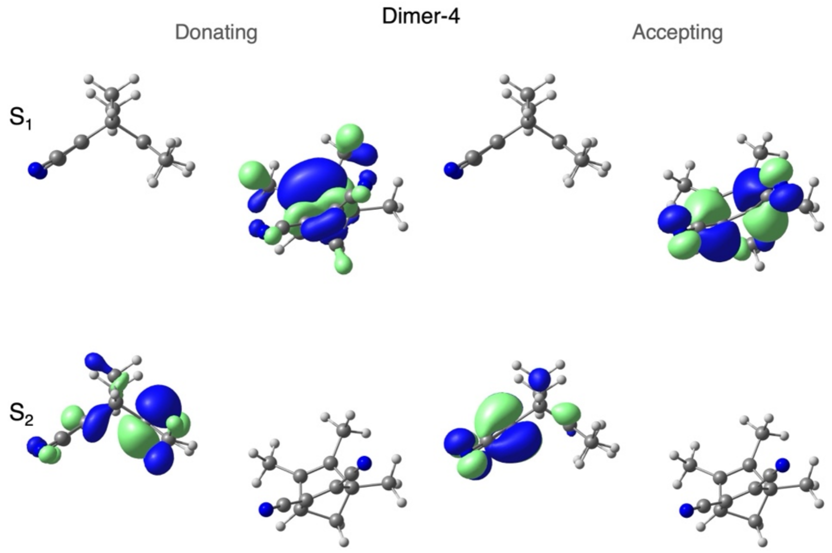

Figure S11. Natural transition orbitals (isovalue = 0.04) of the lowest two transitions of TMDCNBD Dimer-4 in the crystal calculated with LR-TDDFT/ωB97X-D3/aug-cc-pVDZ.

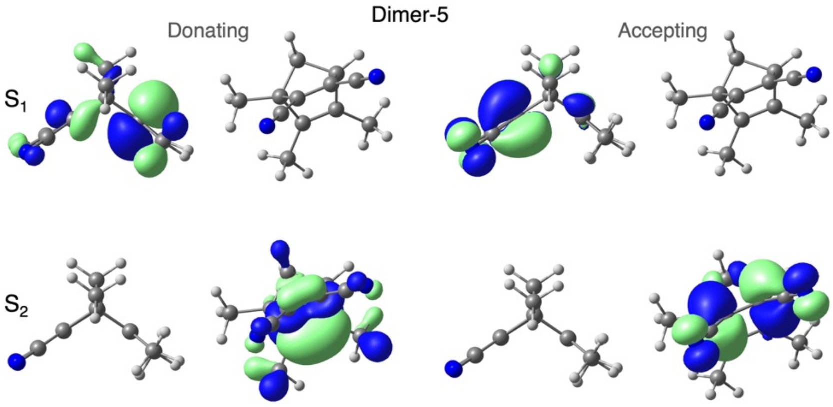

Figure S12. Natural transition orbitals (isovalue = 0.04) of the lowest two transitions of TMDCNBD Dimer-5 in the crystal calculated with LR-TDDFT/ωB97X-D3/aug-cc-pVDZ.

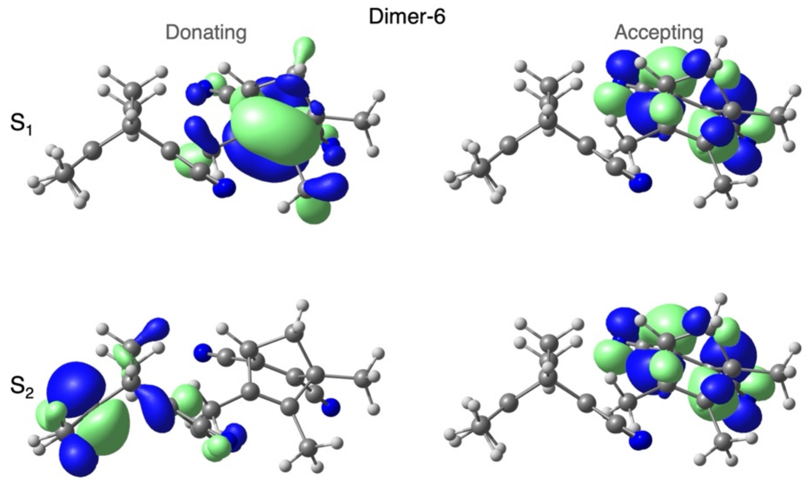

Figure S13. Natural transition orbitals (isovalue = 0.04) of the lowest two transitions of TMDCNBD Dimer-6 in the crystal calculated with LR-TDDFT/ωB97X-D3/aug-cc-pVDZ.

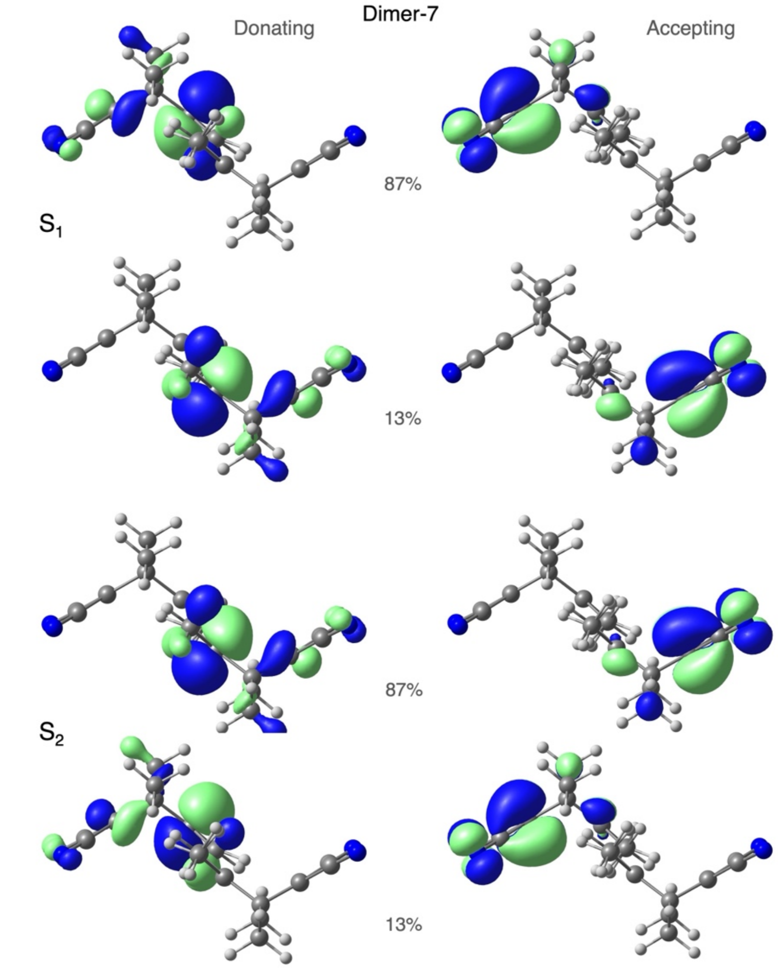

Figure S14. Natural transition orbitals (isovalue = 0.04) of the lowest two transitions of TMDCNBD Dimer-7 in the crystal calculated with LR-TDDFT/ωB97X-D3/aug-cc-pVDZ. The two major contributions per transition are depicted

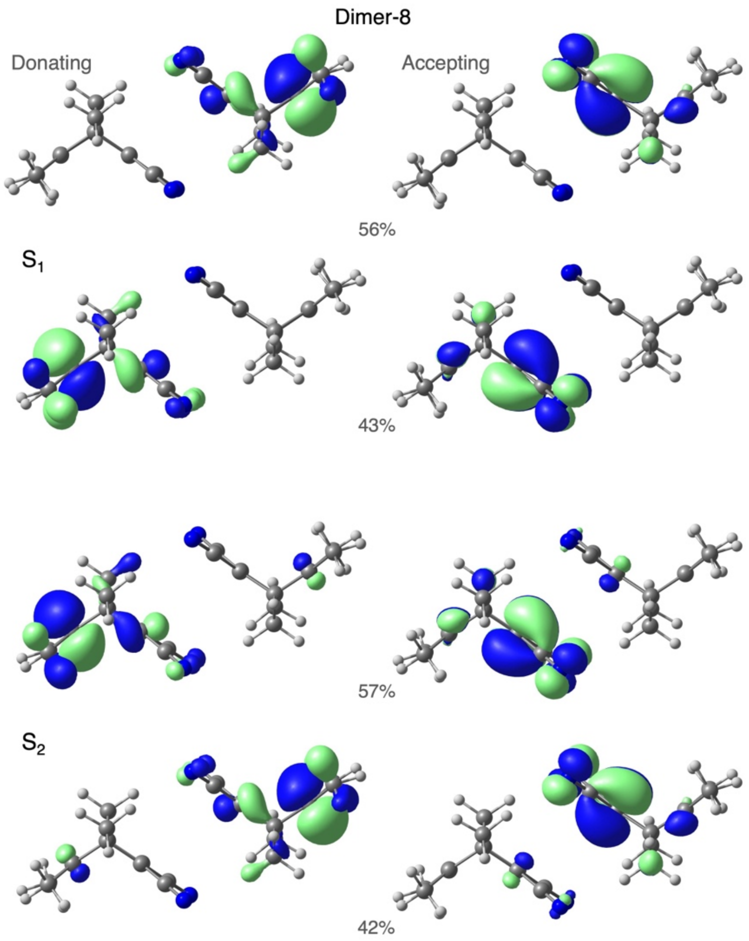

Figure S15. Natural transition orbitals (isovalue = 0.04) of the lowest two transitions of TMDCNBD Dimer-8 in the crystal calculated with LR-TDDFT/ωB97X-D3/aug-cc-pVDZ. The two major contributions per transition are depicted.

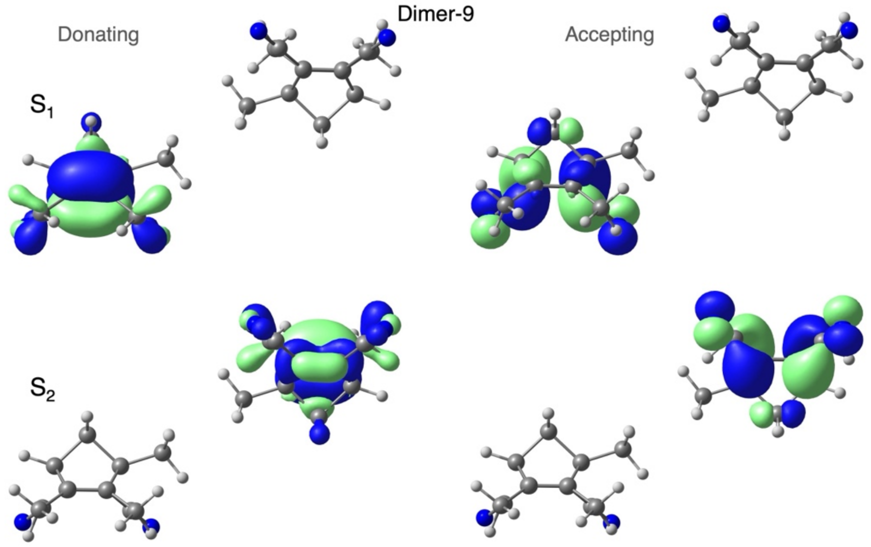

Figure S16. Natural transition orbitals (isovalue = 0.04) of the lowest two transitions of TMDCNBD Dimer-9 in the crystal calculated with LR-TDDFT/ωB97X-D3/aug-cc-pVDZ.

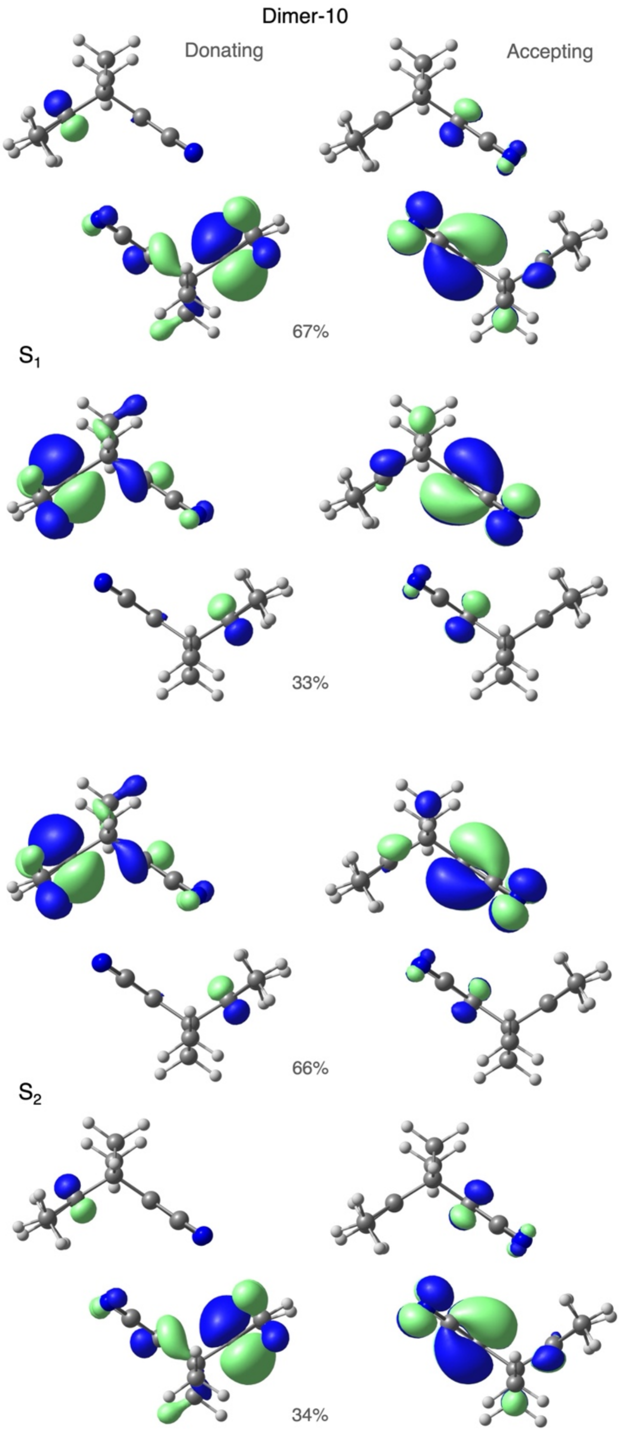

Figure S17. Natural transition orbitals (isovalue = 0.04) of the lowest two transitions of TMDCNBD Dimer-10 in the crystal calculated with LR-TDDFT/ωB97X-D3/aug-cc-pVDZ. The two major contributions per transition are depicted.

S5. **Excited-state dynamics**

The excited-state lifetime was obtained in this work by fitting the total excited-state population (S_1+S_2) using a delayed exponential decay expressed by the following piecewise delayed exponential decay function:

$$f(t) = \begin{cases} 1 & , \ t \leq \tau_1 \\ f_\infty + (1 - f_\infty)e^{-(t-\tau_1)/\tau_2}, & t > \tau_1 \end{cases} \qquad \text{(S1)}$$

where τ_1 is the latency time to initiate the internal conversion, τ_2 is the exponential decay constant, and f_∞ is the fraction of population that has not decayed within this time constant. The lifetime (τ) is then given by $\tau = \tau_1 + \tau_2$. The fitting parameters for the lifetime value shown in Figure 4a are presented in Table S3 below.

Table S3: Parameters used to fit Equation S1 to the total excited-state population decay for the 520 propagated trajectories

Parameter	Value	std dev	unit
f_∞	0.050	0.006	
τ_1	68.5	0.7	fs
τ_2	126.6	2.3	fs

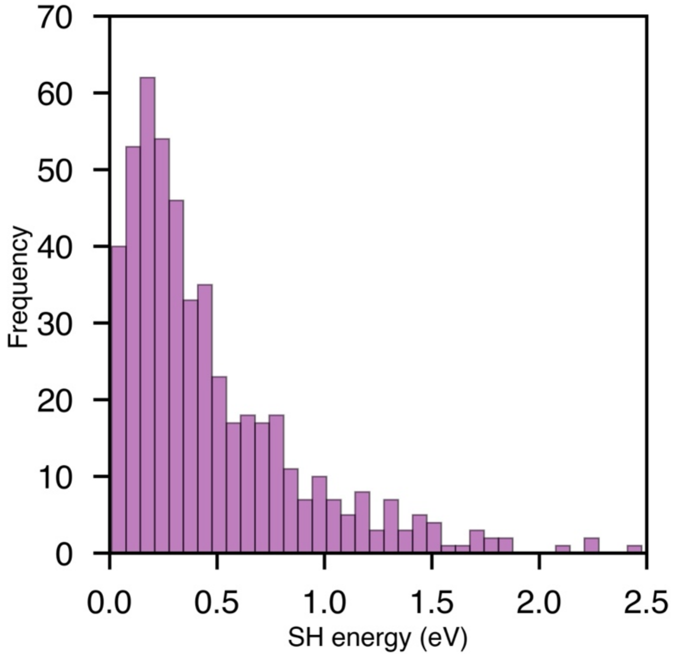

Figure S18. Histogram showing the energy gap between S_1 and S_0 for the $S_1 \rightarrow S_0$ surface hopping process.

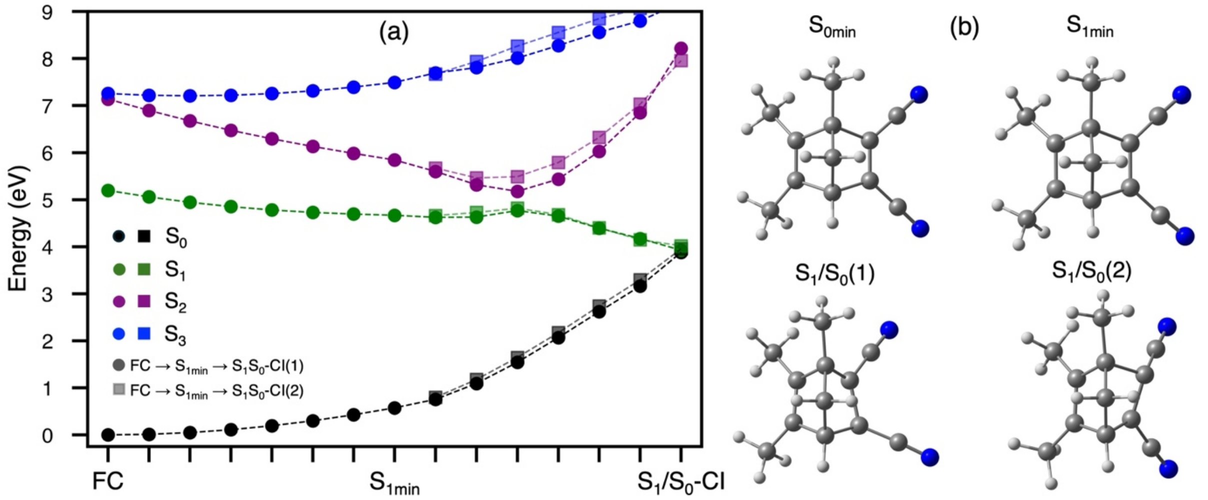

Figure S19. (a) Deactivation pathway for photoexcited TMDCNBD along the interpolated path between the critical points of the potential energy surface FC→S_{1min}→S_1/S_0-CI(1) (circles) and FC→S_{1min}→S_1/S_0-CI(2) (squares). Minima for S_0, S_1 and S_1/S_0-CI(1) and S_1/S_0-CI(2) were obtained at the multiscale level SA(6)CASSCF[8,6]/ANO-S-VDP:GFN2-xTB and connected by a cartesian interpolation path. The four lowest electronic states are depicted. (b) Graphic representation of the minima for the four critical points, S_0, S_1 and S_1/S_0-CI(1) and S_1/S_0-CI(2), considered in the interpolation.

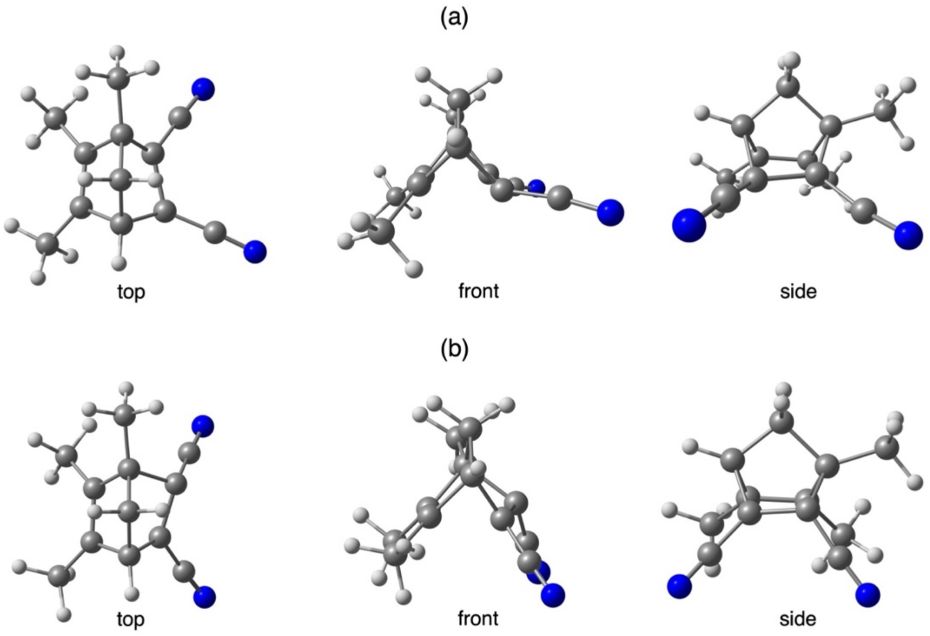

Figure S20. Comparison of two minimum energy CIs (MECIs) optimised within the multiscale OEC model at the SA(6)CASSCF[8,6]/ANO-S-VDP:GFN2-xTB level of theory. Three different views for MECI(1) (a) and MECI(2) (b) are included.

The observed structural change to get to the crossing seam region is dominated by large-amplitude torsional rearrangements along the substituents C-C bonds rather than distortion of the bicyclic core itself. The two optimised MECIs (shown in Figure S20) differ primarily in the three-dimensional orientation of the cyano side chains relative to the norbornadiene framework. In MECI(1), the cyano groups adopt a more extended, outward-oriented conformation depicting a more anti-like arrangement with respect to the bicyclic core. MECI(2), on the contrary, displays the cyano groups folded inward and downward toward the bicyclic scaffold, adopting a significantly more compact and bent conformation. The ultrafast access to the S_1/S_0 crossing region can be rationalised in terms of the vibronic coupling presented in Figure 6 in the manuscript, where the two most active modes involve the ring closing towards the formation of TMDCQC and the torsional distortion of the cyano groups.

In terms of electronic characteristics, both MECIs show charge transfer in the S_1 state from the methylated side to the cyano substituents with a calculated value at the SA(6)CASSCF[8,6]/ANO-S-VDP of 0.38e for CI(1) and 0.25e for CI(2).

We also analysed whether the nonradiative deactivation via the two distinct CIs could drive different photoproducts distribution. However, as we show in Figure S23 and Table S5, comparable distributions of the two main photoproducts are obtained after nonradiative deactivation via surface hopping in the region around $S_1/S_0(1)$ or $S_1/S_0(2)$, thus reinforcing the fact that these two CIs are equivalent not only energetics but also in terms of photoproducts distribution.

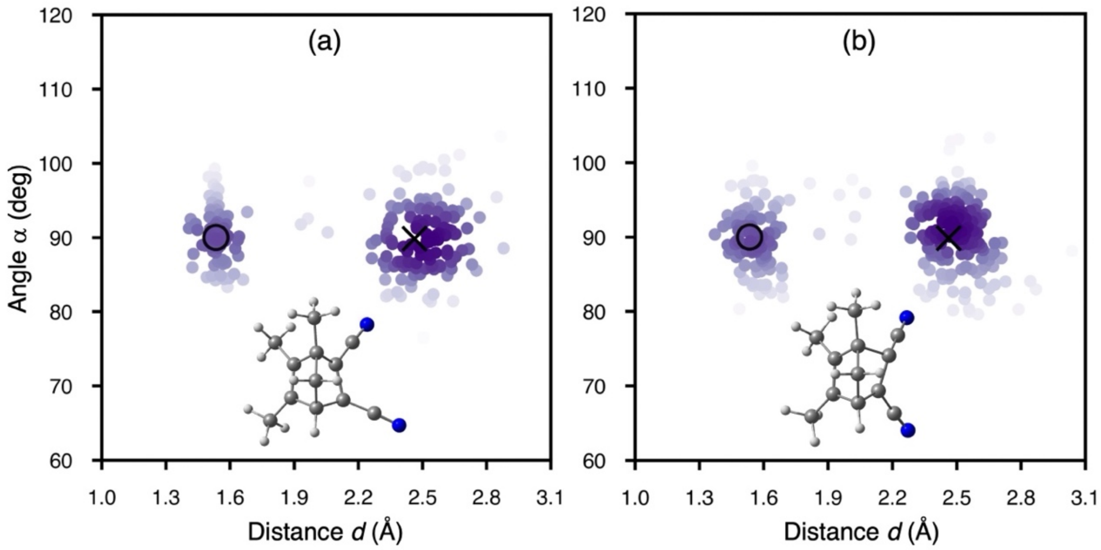

Figure S21. *d* and α distributions of the 198 trajectories (a) and 260 trajectories (b) for the TMDCNBD and TMDCQC photoproduct distribution after deactivation to the ground state via $S_1/S_0(1)$ and $S_1/S_0(2)$, respectively. The intensity of the colours represents the accumulation of the points from low to high, evaluated by Gaussian kernel density estimation. The "**X**" and "**O**" symbols mark the positions of the optimised ground state minimum of TMDCNBD and TMDCQC geometries. The geometries of the two S_1/S_0-MECIs are shown in the insets without displaying the Hydrogens for a better clarity.

Table S4. Photoproducts distribution after deactivating to the ground electronic states via $S_1/S_0(1)$ and $S_1/S_0(2)$

	$S_1/S_0(1)$	$S_1/S_0(2)$
Total number of hops	198	260
Hops generating TMDCNBD	118	149
Hops generating TMDCQC	70	99
Hops generating TMDCPX	6	6
% of TMDCNBD generation	59.6	57.3
% of TMDCQC generation	35.4	38.1
% of TMDCPX generation	3.0	2.3

S6. Energy conservation analysis in the nonadiabatic dynamics

In this section, we present a convergence analysis of the NAMD trajectories, verifying total-energy conservation with an energy drift below 0.25 eV. Figure S22 shows the decay of the total excited-state population together with fits using Equation S1 (fit parameters in Table S5), and Figure S23 shows the evolution of the average π_{C-C} distance, *d*, illustrating the formation of the main photoproducts for trajectory subsets that conserve energy up to 50 fs, 100 fs, 150 fs, and 175 fs. In all cases, the excited-state deactivation time constant τ_{ES} as well as the photoproducts formation quantum yields agree very well with the results presented in the manuscript considering the 520 trajectories.

Table S5: Parameters used to fit Equation S1 to the total excited-state population decay for trajectory subsets ensuring energy conservation up to 50 fs, 100 fs, 150 fs and 175 fs shown in Figure S19.

$E_{conservation}$ time (fs)	Parameter	value	Std dev	unit
50	f_∞	0.050	0.007	
	τ_1	70.8	0.8	fs
	τ_2	116.0	3	fs
100	f_∞	0.050	0.004	
	τ_1	119.5	0.5	fs
	τ_2	83.5	1	fs
150	f_∞	0.061	0.003	
	τ_1	140.4	0.3	fs
	τ_2	72.1	0.7	fs
175	f_∞	0.050	0.003	
	τ_1	155.1	0.3	fs
	τ_2	65.0	0.8	fs

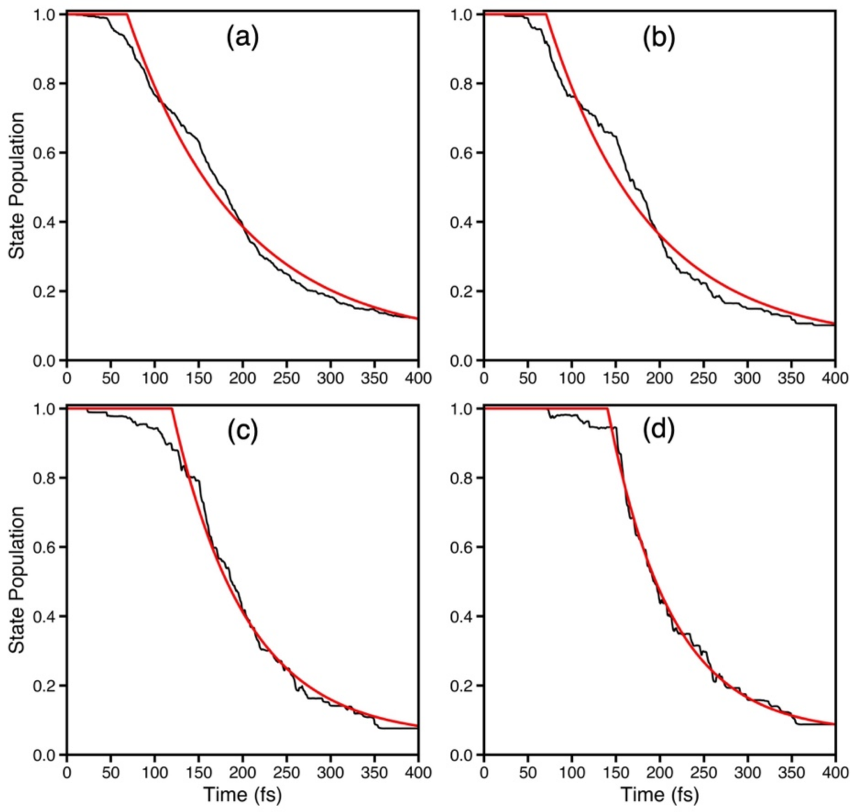

Figure S22. Decay of the total excited-state population (black trace) together with fits using Equation S1 (red trace, fit parameters in Table S5) for trajectory subsets that conserve energy up to 50 fs (a), 100 fs (b), 150 fs (c), and 175 fs (d).

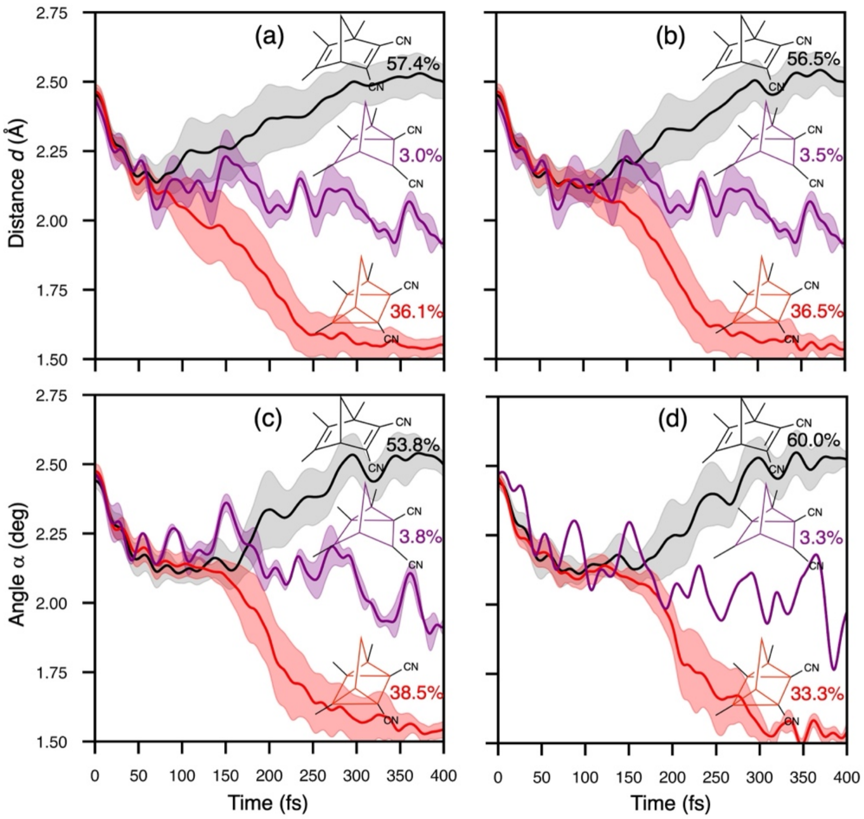

Figure S23. Evolution of the average π_{C-C} distance, *d*, illustrating the formation of the main photoproducts for trajectory subsets that conserve energy up to 50 fs (a), 100 fs (b), 150 fs (c), and 175 fs (d). The Quantum yields obtained for each photoproduct is included in the inset of every f*igure.*

S7. **Electrostatic response of the environment to conformational changes in the central region**

The environment is kept frozen during both the generation of the initial conditions and the nonadiabatic molecular dynamics trajectories. The omission of environmental nuclear and charge fluctuations could, *a priori*, introduce polarisation effects that influence the correct description of both the initial conditions and subsequent dynamics. To address the effect of static charges in the initial sampling, we have calculated different metrics of the environment's electrostatic response to changes in the central region produced by a Wigner distribution (200 structures) around the S_{0min} geometry, or a randomly selected FSSH trajectory conducting from TMDCNBD to TMDCQC (267 structures each obtained every 1.5 fs of dynamics). The electrostatic of the whole cluster considered in our simulations is then obtained with GFN2-xTB, i. e., maintaining the same low-level of theory used throughout the ONIOM scheme considered in this work. The induced polarisation of the environment caused by the sampling generated from the FSSH trajectory is a proxy to the real case as here we are considering the charges of the photoexcited molecule in its ground state potential energy surface rather than within the $S_1 \rightarrow S_0$ deactivation path. Therefore, the polarization of the electronically excited molecule could cause a different effect, particularly in the inner shell of the cluster due to the charge-transfer character of S_1. Nevertheless, the analysis conducted this way allows us to have an estimation of the electrostatic response of the environment when we generate the initial conditions and across the dynamics.

Different metrics were calculated and compared with respect to the point charge distribution obtained at the S_{0min} geometry, considered as the reference (q^{ref}). Figure S24a and S24d depict the normalised probability density of instantaneous charge deviation of the environment, Δq, considering a pointwise ensemble distribution over all cluster atoms and configurations $\Delta q_i(j) = q_i(j) - q_i^{ref}$. Figure S24d and e show the distribution of the root mean square of Δq per atom RMS(Δq) = $\sqrt{\langle \Delta q_i^2 \rangle}$. This measures the response amplitude (polarization strength) of the environment to the fluctuations of the central region. Figure S24c and f show rare events, considered by calculating the distribution of the maximum absolute deviation per atom $\max_j |\Delta q_i(j)|$ within the whole ensemble. This analysis highlights extreme polarisation

events by specifically considering the maximum electrostatic response per atom in the environment across the ensemble of structures considered.

For both sets of sampled structures (Figure S24a-c for Wigner-sampled structures and d-f for the selected FSSH trajectory), we obtained that most of charge deviations $\Delta q_i(j)$ fall in the range below 0.1% with respect to reference values. A small and narrow distribution of RMS(Δq), below 0.25%, and > 99% of extreme events show charge variations below 1%. Overall, we observe a negligible electrostatic response (rigid polarizability) from the environment, even in the extreme events, thus justifying the set of frozen electrostatic environment considered for TMDCNBD in both the initial conditions for the dynamics and across the trajectory towards the formation of TMDCQC. Nevertheless, there are a very few atoms (< 1%) showing extreme charge deviation between 1 and 3% that we analyse below.

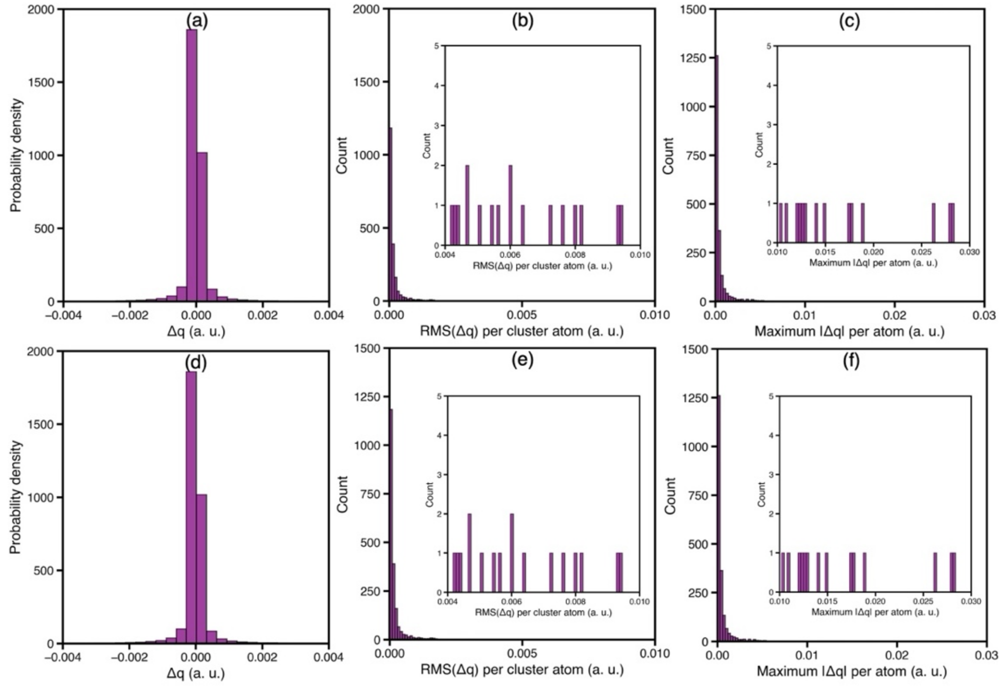

Figure S24. Normalised probability density of instantaneous charge deviation of the environment, Δq, (a and d), distribution of the root mean square of Δq per atom (b and e) and distribution of the maximum absolute deviation per atom $\max_j|\Delta q_i(j)|$ within the whole ensemble (c and f), with respect to the reference set of charges computed at the S_{0min} structure in the crystal. Analysis shown in panels a-c consider 200 Wigner-sampled geometries around the S_{0min} structure, and the analysis in panels d-f consider 267 FSSH sampled geometries for a randomly selected trajectory converting TMDCNBD to TMDCQC.

To get deeper insight in those cases with maximum environment response, we analysed the RMS(Δq) and $\max_j|\Delta q_i(j)|$ resolved by atom type and distance shell, i. e., distance of each atom of the cluster to the centre of mass (COM) of the central molecule, considering both types of ensembles described above (Figure S25). Overall, it is observed that the first neighbour shell (with a response radius ~ 5 – 7 Å of the central TMDCNBD COM) carry most of the electrostatic response. Beyond 7 Å, the environment is effectively electrostatically frozen. The polar π-system C and N atoms are the main carriers of electrostatic response. Although H atoms also contribute, they have a more tightly cluster response, localised in the inner shell (first neighbours). The distribution obtained from the FSSH trajectory is broader compared to the one from Wigner-sampled geometries, extending slightly the environment response distance to COM. This is expected as molecular distortions generated by NAMD (FC → MECI → TMDCQC) are larger than the ones generated via Wigner sampling (around FC).

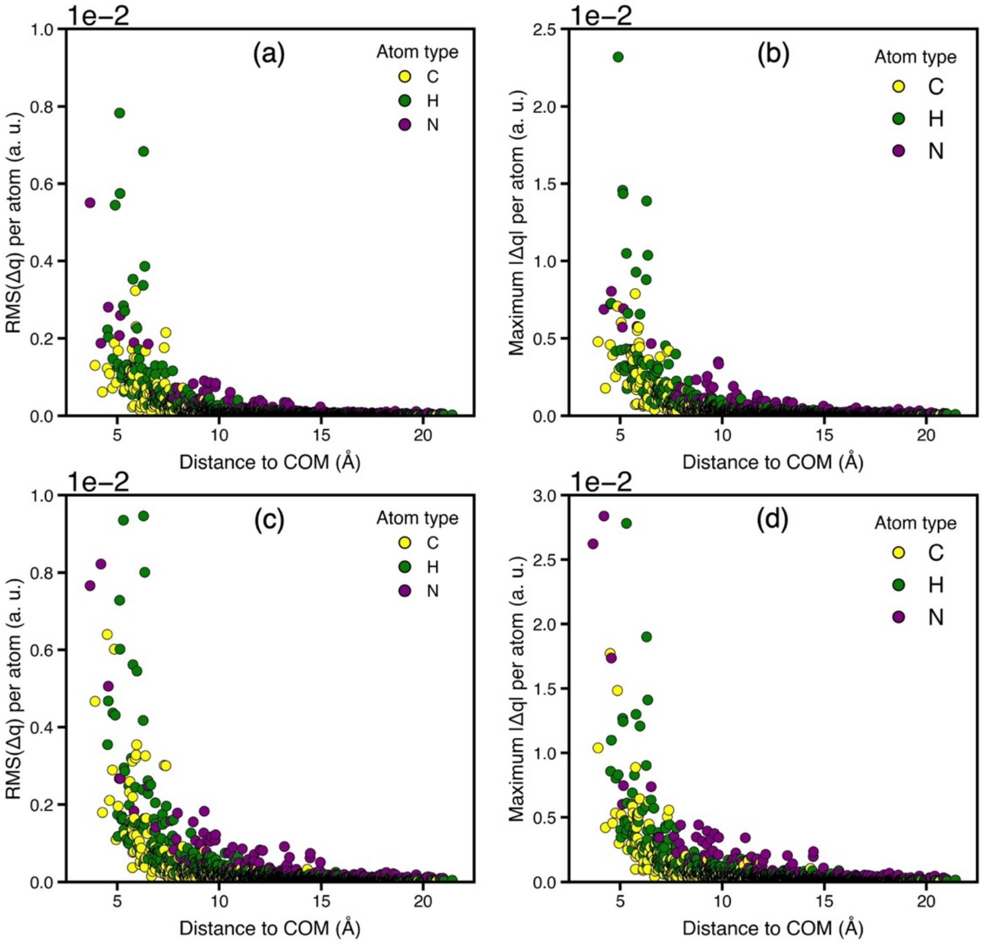

Figure S25. Distribution of the root mean square of Δq per atom (a and c) and distribution of the maximum absolute deviation per atom $\max_j|\Delta q_i(j)|$ within the whole ensemble (b and d), resolved by atom type and distance with respect to the central TMDCNBD centre of mass. Analysis shown in panels a-c consider 200 Wigner-sampled geometries around the S_{0min} structure, and the analysis in panels d-f consider 267 FSSH sampled geometries for a randomly selected trajectory converting TMDCNBD to TMDCQC.

S8. **References**

(1) M. Rivera, M. Dommett, R. Crespo-Otero. *J. Chem. Theory Comput.*, 2019, **15**, 2504–2516.
(2) M. Rivera, M. Dommett, A. Sidat, W. Rahim, R. Crespo-Otero. *J. Comput. Chem.* 2020, **41**, 1045–1058.
(3) J.-D. Chai, M. Head-Gordon. *Phys. Chem. Chem. Phys.*, 2008, **10**, 6615–6620.
(4) R. A. Kendall, T. H. Dunning Jr., R. J. Harrison, *J. Chem. Phys.*, 1992, **96**, 6796-806.
(5) Gi. Li Manni, I. Fdez. Galván, A. Alavi, F. Aleotti, et. al. J. Chem. Theory Comput., 2023, **19**, 6933-6991.
(6) M. J. Frisch, G. W. Trucks, H. B. Schlegel, et. al., *Gaussian 16, Revision A.03*, Gaussian, Inc., Wallingford CT, 2016.
(7) F. J. Hernández, B. F. E. Curchod. *Chem. Commun.* 2025, **61**, 17850-17853
(8) M. Barbatti, M. Bondanza, *R. Crespo-Otero, B. Demoulin, P. O. Dral, G. Granucci, F. Kossoski, H. Lischka, B. Mennucci, S. Mukherjee, M. Pederzoli, M. Persico, M. Pinheiro, Jr, J. Pittner, F. Plasser, E. Sangiogo Gil and L. Stojanovic, J. Chem. Theory Comput., 2022,* ***18****, 6851–6865*
(9) E. D. Glendening, A. E. Reed, J. E. Carpenter and F. Weinhold, NBO Version 3.1, 1998, Theoretical Chemistry Institute, University of Wisconsin, Madison, 1998
